# Coordinated activation of DNMT3a and TET2 in cancer stem cell-like cells initiates and sustains drug resistance in hepatocellular carcinoma

**DOI:** 10.1186/s12935-024-03288-3

**Published:** 2024-03-25

**Authors:** Tao Cheng, Changli Zhou, Sicheng Bian, Kelsey Sobeck, Yahui Liu

**Affiliations:** 1 Department of Hepatobiliary and Pancreas Surgery, First Hospital of Jilin University, Changchun, Jilin 130021 P.R. China; 2 The Hormel Institute, University of Minnesota, Austin, MN 55912 USA; 3 MetroHealth Research Institute, Case Western Reserve University, Cleveland, OH 44109 USA; 4 The Institute on the Biology of Aging and Metabolism, University of Minnesota, Minneapolis, MN 55455 USA

**Keywords:** Hepatocellular carcinoma, Sorafenib, Epigenetics, Drug resistance, DNA methyltransferases, The ten-eleven translocation proteins, DNA methylation, Cancer stem cell-like cells

## Abstract

**Background:**

Resistance to targeted therapies represents a significant hurdle to successfully treating hepatocellular carcinoma (HCC). While epigenetic abnormalities are critical determinants of HCC relapse and therapeutic resistance, the underlying mechanisms are poorly understood. We aimed to address whether and how dysregulated epigenetic regulators have regulatory and functional communications in establishing and maintaining drug resistance.

**Methods:**

HCC-resistant cells were characterized by CCK-8, IncuCyte Live-Cell analysis, flow cytometry and wound-healing assays. Target expression was assessed by qPCR and Western blotting. Global and promoter DNA methylation was measured by dotblotting, methylated-DNA immunoprecipitation and enzymatic digestion. Protein interaction and promoter binding of DNMT3a-TET2 were investigated by co-immunoprecipitation, ChIP-qPCR. The regulatory and functional roles of DNMT3a and TET2 were studied by lentivirus infection and puromycin selection. The association of DNMT and TET expression with drug response and survival of HCC patients was assessed by public datasets, spearman correlation coefficients and online tools.

**Results:**

We identified the coordination of DNMT3a and TET2 as an actionable mechanism of drug resistance in HCC. The faster growth and migration of resistant HCC cells were attributed to DNMT3a and TET2 upregulation followed by increased 5mC and 5hmC production. HCC patients with higher DNMT3a and TET2 had a shorter survival time with a less favorable response to sorafenib therapy than those with lower expression. Cancer stem cell-like cells (CSCs) displayed DNMT3a and TET2 overexpression, which were insensitive to sorafenib. Either genetic or pharmacological suppression of DNMT3a or/and TET2 impaired resistant cell growth and oncosphere formation, and restored sorafenib sensitivity. Mechanistically, DNMT3a did not establish a regulatory circuit with TET2, but formed a complex with TET2 and HDAC2. This complex bound the promoters of oncogenes (i.e., CDK1, CCNA2, RASEF), and upregulated them without involving promoter DNA methylation. In contrast, DNMT3a-TET2 crosstalk silences tumor suppressors (i.e., P15, SOCS2) through a corepressor complex with HDAC2 along with increased promoter DNA methylation.

**Conclusions:**

We demonstrate that DNMT3a and TET2 act coordinately to regulate HCC cell fate in DNA methylation-dependent and -independent manners, representing strong predictors for drug resistance and poor prognosis, and thus are promising therapeutic targets for refractory HCC.

**Supplementary Information:**

The online version contains supplementary material available at 10.1186/s12935-024-03288-3.

## Background

Hepatocellular carcinoma (HCC) represents~90% of all cases of primary liver cancer, and is the second cause of cancer-related death worldwide [1]. Sorafenib is an oral multikinase inhibitor (TKI) that targets several signaling pathways, including the Ras/Raf/MEK/ERK mitogenic signaling pathway, VEGFR signaling pathway and PDGFR signaling pathway. Many patients are present with metastatic disease at diagnosis. Sorafenib is one of the Food and Drug Administration (FDA)-approved first-line systemic therapies for HCC. Despite initial response, many patients display disease progression characterized by acquired sorafenib resistance. As sorafenib inactivates multiple signaling pathways, the acquisition of resistance might involve distinct mechanisms. Indeed, several molecular mechanisms have been proposed to explain how HCC cells avoid sorafenib killing, including transport processes, [2] changes in DNA repair, gene mutations, [3–5] epigenetic aberrations, and dysfunction of compensatory signaling cascades [5]. However, the precise molecular basis underlying acquired sorafenib resistance remains poorly understood.

There are many mechanisms that can lead to drug resistance. In terms of epigenetics, in addition to DNA methylation, there are also mechanisms such as RNA m6A that can cause drug resistance. However, while DNA methylation is one of the most widely studied epigenetic changes, there have been no relevant studies combining the two in the field of liver cancer drug resistance. DNA methylation involves a covalent chemical modification of DNA. On the one hand, in the presence of S- adenosylmethionine, which serves as a methyl donor, a methyl group is added to the C-5 position of cytosine residues yielding 5-methylcytosine (5mC). This DNA methylation process is installed by DNA methyltransferases (mainly DNMT1, DNMT3a and DNMT3b). DNMT1 has been proposed to be the maintenance methyltransferase that transfers the methylation patterns to daughter cells, [6] but DNMT3a and DNMT3b perform *de novo* methylation on unmethylated CpG dinucleotides [7]. On the other hand, 5mC can be the substrate of the ten-eleven translocation proteins (TET1, TET2, TET3)-mediated oxidation process. This leads to the formation of 5-hydroxy-methylcytosine (5hmC), promoting locus-specific reversal of DNA methylation [8]. When dysregulated or/and mutated, DNMTs and TETs break the balance between 5mC and 5hmC production, leading to aggressive cancer growth [9, 10]. Notably, loss-of-function TET2 mutations are frequent in leukemia, [11, 12] but much low mutation rate has been observed in HCC (< 5%).^13^ Although all three TETs are considered to be tumor suppressors in various cancers, evidence is emerging that TET1 and TET2 upregulation has oncogenic potential in certain cancer types, like HCC, [13] leukemia [14] and breast cancer [15]. Mechanistically, aberrant epigenetic changes have been appreciated in HCC tumorigenesis [16] and drug resistance in other cancers, [9, 17] but the underlying mechanisms have been poorly defined so far. Moreover, DNMTs and TETs are scaffold proteins that recruit other transcription factors to regulate target expression, but it is largely unknown whether and how such regulatory activities promote resistance to sorafenib in HCC.

In this study, we explored the role of DNMT3a in coordination with TET2 in promoting HCC cell resistance to sorafenib, as well as the mechanism by which DNMT3a and TET2 regulate resistance-sustaining genes. We demonstrate that functional cooperation of DNMT3a and TET2 is required for HCC cells to escape sorafenib killing. Mechanistically, upregulation of DNMT3a and TET2 further silences TSGs through promoter DNA hypermethylation; In parallel, TET2 did not have a regulatory feedback loop with DNMT3a, but forms a complex with DNMT3a and HDAC2. This complex bind oncogene promoters and induces oncogenic upregulation in resistant cells. Both genetic and pharmacological inactivation of DNMT3a or/and TET2 impairs-sorafenib resistant (sorafenibR) cell growth. Our discoveries demonstrate DNMT3a and TET2 coordination as robust targets, whose disruption represents a promising strategy to increase the therapeutic efficacy of sorafenib in patients with refractory HCC.

## Methods

### Cell lines, cell culture, vectors and reagents

The Hep3b and Huh7 cell lines were obtained from American Type Culture Collection with no further authentication or testing for mycoplasma. The cell lines were grown in MEM (Hep3b, CORNING #10-010-CV) or DMEM (Huh7, CORNING #10-013-CV), supplemented with 10% FBS (Gibco by Life Technologies™, #16140-071) and Antibiotic-Antimycotic (Gibco by Life Technologies™, #15,240,062) at 37 °C under 5% CO_2_. No cell line used in this paper is listed in the database of commonly misidentified cell lines maintained by ICLAC (International Cell Line Authentication Committee). The shRNAs against DNMT3a (V2LHS_202509, V2LHS_74666, V2LHS_74668), TET2 (V2LHS_227656, V2LHS_266084) and the negative control (pGIPZ) vectors were obtained from BMGC RNAi (University of Minnesota). TET2 inhibitor Bobcat339 (#408,006) was obtained from MedKoo Biosciences, and Sorafenib (#SML2653) was obtained from Sigma-Aldrich. Solutions of Bobcat339 were prepared in dimethyl sulfoxide (DMSO) at 100 mM, while Solutions of Sorafenib were prepared in DMSO at 10 mM.

### In vitro adaption of sorafenib-resistant (sorafenibR)cells

Hep3b and Huh7 cell lines were passaged with low concentration of sorafenib (0.1 µM) and sequentially cultured in increasing concentrations of sorafenib (0.3, 1, 2 µM) for 8 weeks. Cells cultured in parallel without drugs (DMSO) served as parental negative controls. Cells were considered resistant when they could routinely grow in respective medium containing 2 µM sorafenib.

### Lentivirus production and infection for gene knockdown

For virus production, about 3.8 × 10^6^ HEK-293 cells were plated in a 10 cm cell culture dish. After 24 h, cells were transfected with 6 µg of target plasmids or scrambled control plasmids using calcium phosphate transfection reagent (CalPhos™ Mammalian Transfection Kit). The lentiviruses were harvested at 48 and 72 h after transfection and concentrated according to the protocol of the Lenti-X™ Concentrator (#631,232, Clotech). For virus infection, about 1 × 10^6^ HCC cells were infected by the lentiviruses using Polybrene (final concentrate 4 ug/ml) in 1 ml respective medium and Puromycin (final concentration 2 µg/ml) was added to select the stable transformants 24 h post-infection.

### Cell proliferation assays

Hep3b or Huh7 cells with various treatments were seeded into a 24-well or 12-well culture plates for 24 h. Then the plates were incubated in the IncuCyte S3 Live-Cell Analysis System for real-time analysis. Data were analyzed by the software provided. The total area of phase or green fluorescent cells for each picture was calculated, which represents the confluence of Hep3b or Huh7 cells.

### Wound healing assays

Cells with various treatments were first seeded into a 96-well culture plate, then subjected to wound healing assays on the 2nd day. The plates were then put into the IncuCyte S3 Live-Cell Analysis System for real-time analysis. Cell migration toward the wound was photographed under IncucyteS3, and the migration distance was assessed by CorelDRAWX5 Software.

### Apoptosis and cell cycle assays

Cell apoptosis and cell cycle assays were performed according to the manufacturer’s instruction using Annexin V-PI Apoptosis Detection Kit I (#556,547, BD Pharmingen™) and CycleTEST PLUS DNA Reagent Kit (#340,242, BD Pharmingen™), respectively, followed by flow cytometry analysis.

### CCK-8 assays

CCK-8 assays were performed using Cell Counting Kit-8 (CCK-8, Dojindo Molecular Technologies, #CK04-11). Briefly, parental and resistant Hep3b or Huh7 cells (1.5 × 10^4^) in MEM or DMEM medium (100 µl) were dispensed into 96-well flat-bottomed microplates and drugs were added after 24 h of incubation. The cells were cultured for additional 24 or 48 h, and CCK-8 reagent (10 µl) was added to each well. Then the microplates were incubated at 37 °C for another 1~2 h. Absorbance was measured at 450 nm using a microplate reader and the results were expressed as a ratio of treated to untreated cells (as 100%). Four wells were sampled per each experimental group in a given experiment. Averages are reported ± SD.

### Oncosphere-forming assays

About 1,000 Hep3b or Huh7 cells were seeded in appropriate medium in each well of a Corning® Costar® Ultra-Low attachment plate (#3473) and incubated for about 7 days. During the incubation, the cells were pipetted every 2 days. Then the number of oncospheres was counted and images were taken.

### DNA dotblotting

Genomic DNA was extracted using DNA Blood/Tissue Kit (QIAGEN), denatured and subjected to dotblotting analysis using antibodies against 5mC or 5hmC (Supplementary Table 2) as previously described [18, 19]. The DNA spotted membrane was stained with 0.02% methylene blue (Sigma) in 0.5 M sodium acetate (pH 5.0) for DNA loading control.

### Promoter methylation assays

Genomic DNA was isolated from parental and sorafenib^R^ HCC cells or sorafenib^R^ Hep3b cells with knockdown of TET2 or DNMT3a gene, or with treatment of Bobcat339. The DNA (1 µg) from these cells was digested by HpaII or BstUI for 2–4 h. The digested DNA was cleaned by PCR purification kit (QIAGEN) and subjected to SYBR Green PCR using primers specific for TSG promoters (CpG enriched region; Supplementary Table 1).

### Co-immunoprecipitation (Co-IP) and Western blotting

Whole cellular lysates were prepared by harvesting the cells in 1 × cell lysis buffer (20 mM HEPES (pH 7.0), 150 mM NaCl and 0.1% NP40) supplemented with 1 mM phenylmethane sulfonyl fluoride (PMSF, #10,837,091,001, Sigma), 1 × Phosphatase Inhibitor Cocktail 2 and 3 (Sigma, #P5726, P0044), and 1 × protease inhibitors (protease inhibitor cocktail set III, Calbiochem-Novabiochem, #539,134). Approximately 0.5-1 mg total protein lysate was precleared with 70 µL of slurry of protein G Dynabeads (Thermo Fisher, #10004D) for 2 h at 4 °C. Dynabeads (70 µl) were coated with 2 to 5 µg of respective antibodies at 4 °C overnight. The dynabeads were washed in 1 x cell lysis buffer and boiled in 1 x loading buffer for 5–10 min to elute proteins. The immunoprecipitants and whole cell lysates were subjected to Western blotting using our established methods [18, 20, 21]. The Western blots were quantified using the Image J Software from the U.S. National Institutes of Health. The antibodies used are listed in Supplementary Table 2.

### Chromatin immunoprecipitation (ChIP)

ChIP assays were performed using the Chromatin Immunoprecipitation Assay Kit (Millipore Sigma, #17–295) according to the manufacturer’s standard protocol. DNA was quantified using qRT-PCR with SYBR green incorporation (Applied Biosystems). The antibodies used are listed in Supplementary Table 2. The primers for gene expression are listed in Supplementary Table 1.

### RNA extraction, cDNA preparation, quantitative PCR (qPCR)

RNA was extracted using the miRNeasy Kit (QIAGEN) and reverse transcription for cDNA was performed using the High-Capacity cDNA Reverse Transcription Kit (#4,368,814, Applied Biosystems). The SYBR-Green qPCR (#4,309,155, Applied Biosystems) was used to measure gene expression. *18 S* levels were used for normalization and target expression was analyzed using the ΔCT approach. The primers are listed in Supplementary Table S1.

### Methylated DNA immunoprecipitation (MeDIP) assays

MeDIP assays were performed using the MeDIP Assay Kit (#55,009, Active Motif) according to the manufacturer’s standard protocol. Briefly, genomic DNA was extracted using the DNA Blood/Tissue Kit, and 10–20 ug DNA was sonicated to obtain fragments ranging from 200 to 500 bp. The sonicated DNA fragments were mixed with 5-mC antibody and magnetic beads in IP incubation mix overnight at 4 °C on a rotating wheel. Beads were then washed three times with wash buffer 1 and once with 100 µL of ice-cold wash buffer 2. The supernatant was discarded, and bead pellets were preserved for DNA elution. Two immunoprecipitation (IP) and two input samples were immunoprecipitated per MeDIP array. qPCR was used to quantify the enriched DNA for both Input and IP samples. MeDIP efficiency using qPCR was calculated using following formula: %(meDNA-IP/Total input) = 2^[(Ct(10%input)-3.32) – Ct(meDNA-IP)] x 100%.

### Analysis of gene expression omnibus (GEO) data analysis

GSE109211 was downloaded from the Gene Expression Omnibus (GEO) datasets and analyzed for the expression of DNMTs and TETs, as well as for survival analysis. The samples were normalized, managed, and analyzed using GraphPad Prism 8 Software, employing the Log-rank test and online tools (Gepia2).

### Statistical analysis

The statistical analysis was performed using the Student’s t test. All analyses were performed using the GraphPad Prism 8 Software. P values < 0.05 were considered statistically significant. All p values were two-tailed. All criteria were pre-established. No statistical method was used to predetermine sample size and the sample size for all experiments was not chosen with consideration of adequate power to detect a pre-specified effect size. In vitro experiments, such as qPCR, Western blotting, dotblotting, cell proliferation and apoptosis assays, and wound-healing assays were routinely repeated three times unless indicated otherwise in figure legends or the main text. For each figure, the statistical tests were justified as appropriate.

## Results

### Sorafenib^R^ HCC cells display improved viability, enhanced migration and reduced apoptosis after transient sorafenib treatment

**Fig. 1:**
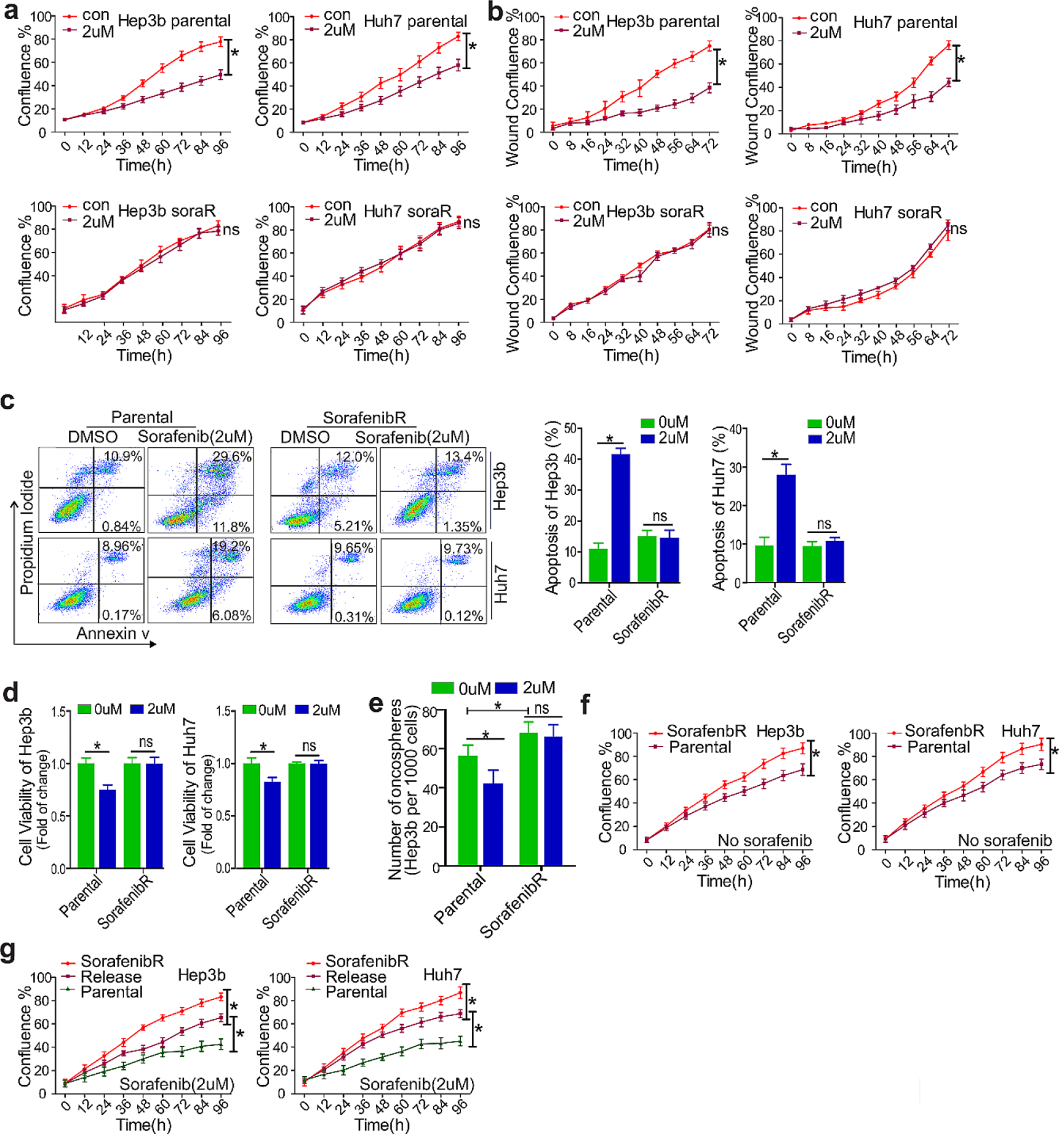
Characterization of sorafenib resistant phenotypes. a and b, Parental and resistant Hep3b or Huh7 cells were transiently exposed to 2 µM sorafenib for indicated time points and subjected to proliferation (**a**) and wound-healing (**b**) assays using IncuCyte proliferation/migration assay. Data represents two independent experiments. (**c**), Parental and resistant Hep3b or Huh7 cells were transiently treated with 2 µM sorafenib for 72 h, and flow cytometry was used to measure apoptotic cells shown as percentage change. Graphs are the quantification of apoptotic cells shown as mean values ± S.D. from three independent experiments. (**d**), CCK-8 assays were used to measure cell viability upon exposure of parental and resistant Hep3b or Huh7 cells to 2 µM sorafenib for 72 h. Data represents two independent experiments with 8 repeats in total. (**e**), Oncosphere assays in parental and resistant Hep3b cells growing in medium with or without drug. Data represents three independent experiments. (**f**), IncuCyte proliferation assay in parental, resistant Hep3b or Huh7 cells growing in drug free medium (**g**), IncuCyte proliferation assays in parental, resistant or release (resistant cells growing in drug free medium for 14 days) Hep3b or Huh7 cells treated with sorafenib for indicated time points. *P < 0.05; ns, not significant; Con, control

To gain insights into the mechanisms of sorafenib resistance, we initially modeled the response of HCC Hep3b and Huh7 cells to sorafenib by continuously culturing them with a stepwise increase of drug dosages for 8 weeks. The final concentration of sorafenib was 2 µM, which exerted sufficient inhibitory action and was in the range of clinically achievable levels [22, 23]. To characterize these sorafenib-selected cells, we first measured the proliferation of parental and resistant (sorafenib^R^) cells upon transient exposure to 2 µM sorafenib. Although all parental controls displayed significant decreases in cell proliferation, the sorafenib^R^ cells did not show an obvious difference in cell proliferation (Fig. 1a and Fig. S1a), upon exposure to sorafenib. This was further confirmed by the enhanced wound-healing capability in sorafenib^R^ versus parental Hep3b and Huh7 cells (Fig. 1b and Fig. S1b). Second, flow cytometry analysis revealed that transient sorafenib treatment does not induce obvious alterations in cell apoptosis and cell viability in sorafenib^R^ Hep3b and Huh7 cells, but significantly promotes cell apoptosis and impairs cell viability in parental controls (Fig. 1c and d). We did not see obvious changes in cell cycles in both parental and sorafenib^R^ Hep3b and Huh7 cells when exposed to 2 µM sorafenib (Fig. S1c). Third, we observed that, as compared with parental cells, oncosphere formation is enhanced in sorafenib^R^ cells that are less affected by sorafenib treatment (Fig. 1e; Fig. S1d). Sorafenib^R^ cells also exhibit a higher rate of cell proliferation in drug-free medium (Fig. 1f; Fig. S1e). These findings are in line with our previous data showing that lung cancer cells resistant to tyrosine kinase inhibitors (i.e., midostaurin) migrate and grow at a faster pace than parental cells in vitro, and possess higher tumorigenic potential in vivo [9]. 

As sorafenib discontinuation is a frequent event in HCC patients, we mimicked sorafenib “holiday” by culturing sorafenib^R^ cells in a drug-free medium for 14 days (released cells). Interestingly, the released cells displayed proliferation impairment, which is supported by a reduction of cell proliferation, and increase of cell apoptosis in the presence of sorafenib, when compared with parental and sorafenib^R^ cells (Fig. 1g; Fig. S1f). These results indicate that partial re-acquisition of sensitivity to sorafenib leads to a transient proliferation arrest upon drug withdrawal, phenocopying the drug holiday effect seen clinically. This is consistent with our recent findings in leukemia that resistance to TKIs (i.e., nilotinib, imatinib) displays reversible features or a transient proliferation arrest upon exposure to the same drug [20]. Finally, we performed sequencing for epigenetic regulators, for example, DNMT3a and TET2 that are frequently mutated in cancers, in sorafenib^R^ Hep3b and Huh7 cells. We did not find any acquired mutations in these genes. Taken together, these results suggest that non-genetic mechanisms could be essential in sorafenib resistance, and although generated in vitro, our drug-resistant HCC cell line derivatives faithfully recapitulate clinical drug resistance.

### HCC cells with sorafenib-acquired resistance have activation of pathways involved in DNA methylation

**Fig. 2:**
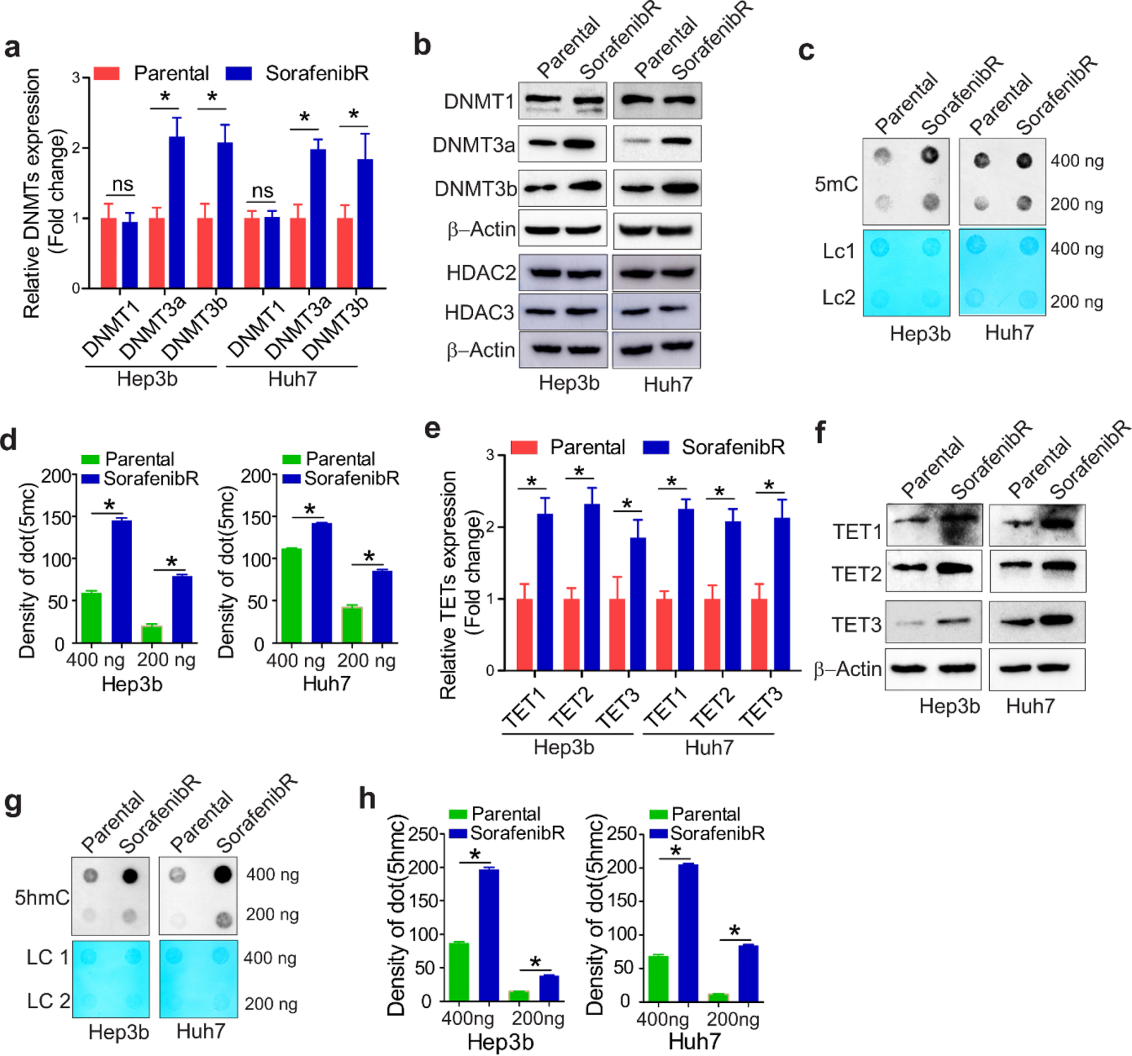
Sorafenib^R^ and parental Hep3b/Huh7 cells show different DNA methylation potential. a and b, qPCR (**a**) or Western blotting (**b**) measuring expression levels of DNMT1, DNMT3a, DNMT3b, HDAC2 and HDAC3. Data are shown as mean values ± SD. c and d, Dotblotting measuring changes in DNA methylation using 5mC antibody (**c**). The graphs (**d**) show the quantification of dot intensity as mean values ± S.D. from three independent experiments. e and f, qPCR (**e**) or Western blotting (**f**) measuring expression levels of TET1, TET2, and TET3. Data are shown as mean values ± SD. g and h, Dotblotting measuring changes in DNA hydroxymethylcytosine using 5hmC antibody (**g**). The graphs (**h**) show the quantification of dot intensity as mean values ± S.D. from three independent experiments. *P < 0.05; ns, not significant; LC, loading control

To identify critical molecules that promote sorafenib resistance, we examined the expression of DNA methyltransferases (DNMTs) in parental and sorafenib^R^ Hep3b and Huh7 cells, because epigenetic aberration (i.e., DNA hypermethylation) is becoming increasingly important in the development of TKI resistance [9, 20]. The results from qPCR and Western blotting disclosed that expression of DNMT3a and DNMT3b, two *de novo* DNA methyltransferases, [7] is increased at both RNA and protein levels. However, expression of maintenance DNMT1, [24] as well as histone protein modifiers HDAC2 and HDAC3, is not obviously changed (Fig. 2a and b). As DNA methylation is installed by DNMTs via adding a methyl group to the C-5 position of cytosine residues to form 5-methylcytosine (5mC), we proceeded to determine whether the mount of 5mC a is changed in sorafenib^R^ cells. DNA dotblotting by the anti-5mC antibody [10, 25] revealed that 5mC production is significantly elevated in sorafenib^R^ Hep3b and Huh7 cells compared with parental counterparts (Fig. 2c and d). These results expand previous findings showing that 5mC amount is markedly increased in leukemia cells resistant to nilotinib [10] and in lung cancer cells resistant to midostaurin [9]. 

It has been shown that TET methylcytosine dioxygenases catalyze the conversion of 5mC to 5hmC, resulting in DNA demethylation [8, 26–28]. To further understand the role of DNA methylation in sorafenib resistance, we performed qPCR and Western blot for changes of TETs, and found that expression of TET1, TET2 and TET3 is significantly upregulated at both RNA and protein levels in sorafenib^R^ cells compared with parental cells (Fig. 2e and f). Dotblotting analysis using anti-5hmC antibody revealed that 5hmC level is much higher in sorafenib^R^ Hep3b and Huh7 cells than that in parental counterparts (Fig. 2g and h), in agreement with the methylcytosine dioxygenase activities of TET1, TET2 and TET3. Collectively, these findings suggest that long-term exposure to sorafenib alters the functions of DNA methylation machinery in HCC cells.

### Knockdown of DNMT3a and TET2 impairs sorafenib^R^ HCC cell growth

**Fig. 3:**
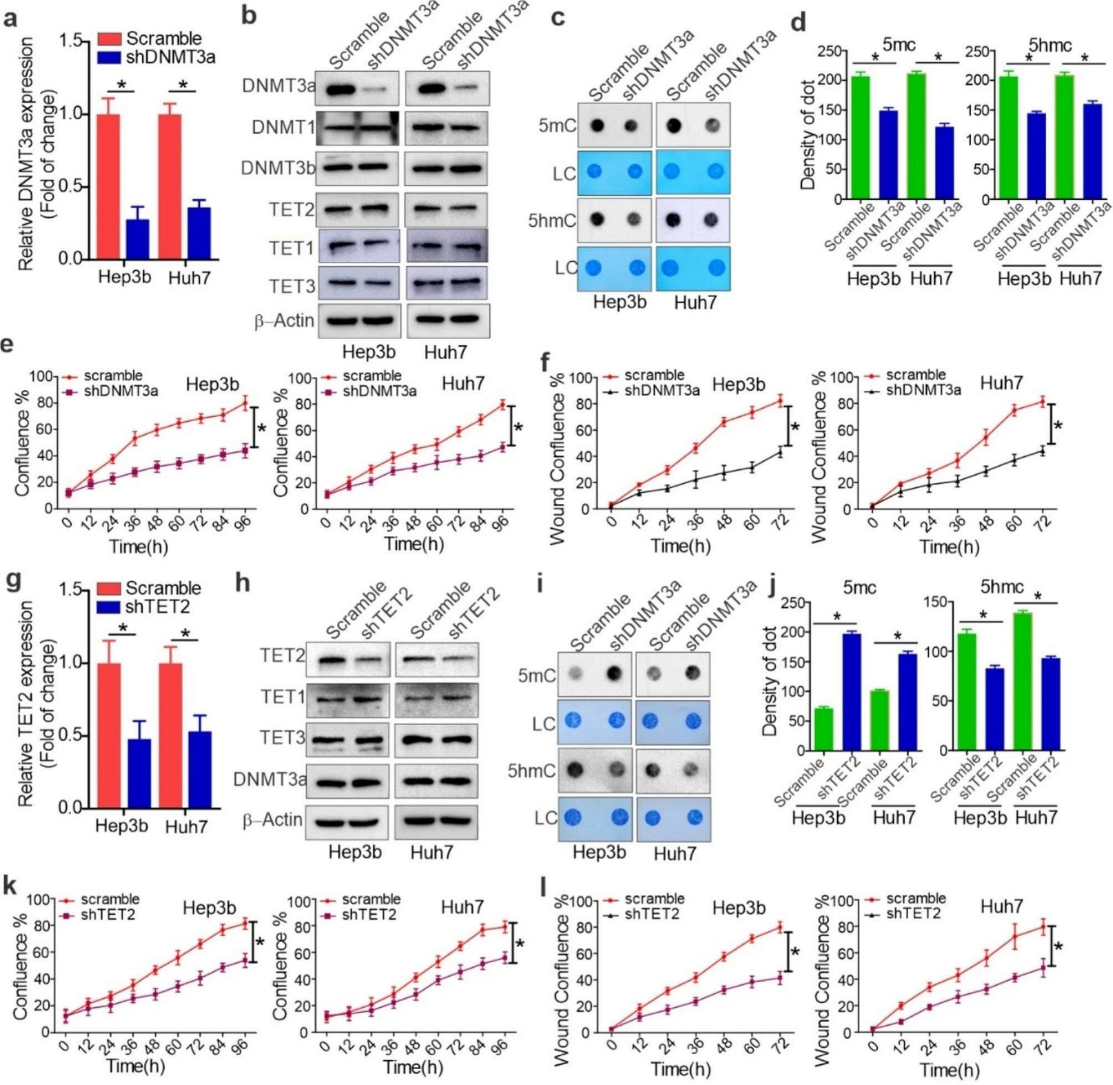
Role of DNMT3a and TET2 in resistant cell growth. Sorafenib^R^ Hep3b and Huh7 cells were infected with DNMT3a or TET2 shRNA or control viruses and selected by 2 µg/ml puromycin for 5 days. **a** and **b**, qPCR (**a**) and Western blotting (**b**) assessing efficacy of DNMT3a knockdown. Data represents three independent experiments, and the graphs are shown as mean values ± S.D. **c** and **d**, Dotblotting measuring changes of DNA methylation or hydroxymethylcytosine using 5mC and 5hmC antibodies. The graphs (**d**) are the quantification of dot intensity shown as mean values ± S.D. from three independent experiments. **e** and **f**, Proliferation (**e**) and migration (**f**) assays in resistant cells 5 days after virus infection in drug-free medium. Data represents two independent experiments with 12 repeats in total. **g** and **h**, qPCR (**g**) and Western blotting (**h**) showing TET2 knockdown. Data represents three independent experiments. **i** and **j**, Dotblotting measuring changes in DNA methylation or hydroxymethylcytosine using 5mC and 5hmC antibodies (**i**). The graphs (**j**) are the quantification of dot intensity as mean values ± S.D. from three independent experiments. **k** and **l**, Proliferation (**k**) and migration (**l**) assays in resistant cells 5 days after virus infection in drug-free medium. Data represents two independent experiments with 12 repeats in total. *P < 0.05; ns, not significant

Having demonstrated the upregulation of a *de novo* DNA methyltransferase DNMT3a [7], in sorafenib^R^ HCC cells, next we sought to determine the biological functions of DNMT3a aberrations. Sorafenib^R^ Hep3b and Huh7 cells were infected with DNMT3a shRNA or scrambled vectors for 24 h and further selected by 2 µM puromycin for an additional 96 h. First, the efficacy of virus infection was verified by the high rate of GFP/fluorescent cells (Fig. S2a). The shRNA-3, which showed the most DNMT3a reduction, was used for further investigations. As shown in Fig. 3a and b, both RNA and protein expression of DNMT3a was markedly decreased in DNMT3a shRNA compared with the control group. The specificity of DNMT3a knockdown was supported by the unchanged expression of DNMT1, DNMT3b, TET2, TET1 and TET3 in DNMT3a shRNA versus control cells. Second, the results from dotblotting showed that 5mC production is much lower in DNMT3a-depleted cells than that in control groups (Fig. 3c and d). Interestingly, DNMT3a knockdown led to a decrease 5hmC levels even with unchanged TET2 protein levels. This may have resulted from a reduction of 5mC, the original substrate of TET2. Third, DNMT3a depleted cells were subjected to proliferation and wound-healing assays. We observed that cells with DNMT3a knockdown could proliferate and migrate at a much lower rate, in a time-dependent manner, than their control counterparts (Fig. 3e and f; Fig. S2a and Fig. S2b). These findings support an important contribution of DNMT3a to sorafenib^R^ cell growth.

Having shown that TET2 is upregulated in sorafenib^R^ compared with parental cells, next we sought to examine whether TET2 expression is essential for sorafenib^R^ cell growth. To this end, sorafenib^R^ Hep3b and Huh7 cells were infected with TET2 shRNA or control vectors for 24 h followed by puromycin selection for an additional 96 h. The high rate of GFP/fluorescent cells indicated the high efficacy of virus infection (Fig. S2c). The results from qPCR and Western blot demonstrated efficient knockdown of the TET2 gene, and the specificity of TET2 knockdown was supported by unchanged levels of TET1, TET3 and DNMT3a in TET2 shRNA-transfected cells (Fig. 3g and h). Because TET2 is a methylcytosine dioxygenase, we speculated that TET2 ablation might decrease 5hmC production. Indeed, the results from dotblotting using 5hmC antibody support that sorafenib^R^ Hep3b and Huh7 cells have much lower levels of 5hmC than parental cells (Fig. 3i and j). Notably, TET2 knockdown led to an increase in 5mC abundance even when DNMT3a protein expression remained unchanged. This may be attributed to the downregulation of TET2, leading to less conversion of 5mC to 5hmC.

To investigate the biological outcomes of TET2 knockdown, sorafenib^R^ Hep3b and Huh7 cells with TET2 depletion were subjected to proliferation and wound-healing assays. We found that, compared to those with scrambled controls, cells with TET2 knockdown proliferate at a much slower rate (Fig. 3k, Fig. S2c) and migrate a much shorter distance (Fig. 3l, Fig. S2d), which occurs in a time-dependent manner. These findings suggest that TET2 is required for the aggressive proliferation and migration of sorafenib^R^ HCC cells.

### Cancer Stem cells (CSCs) with upregulation of DNMT3a and TET2 are more tolerant to sorafenib-induced cell death

**Fig. 4:**
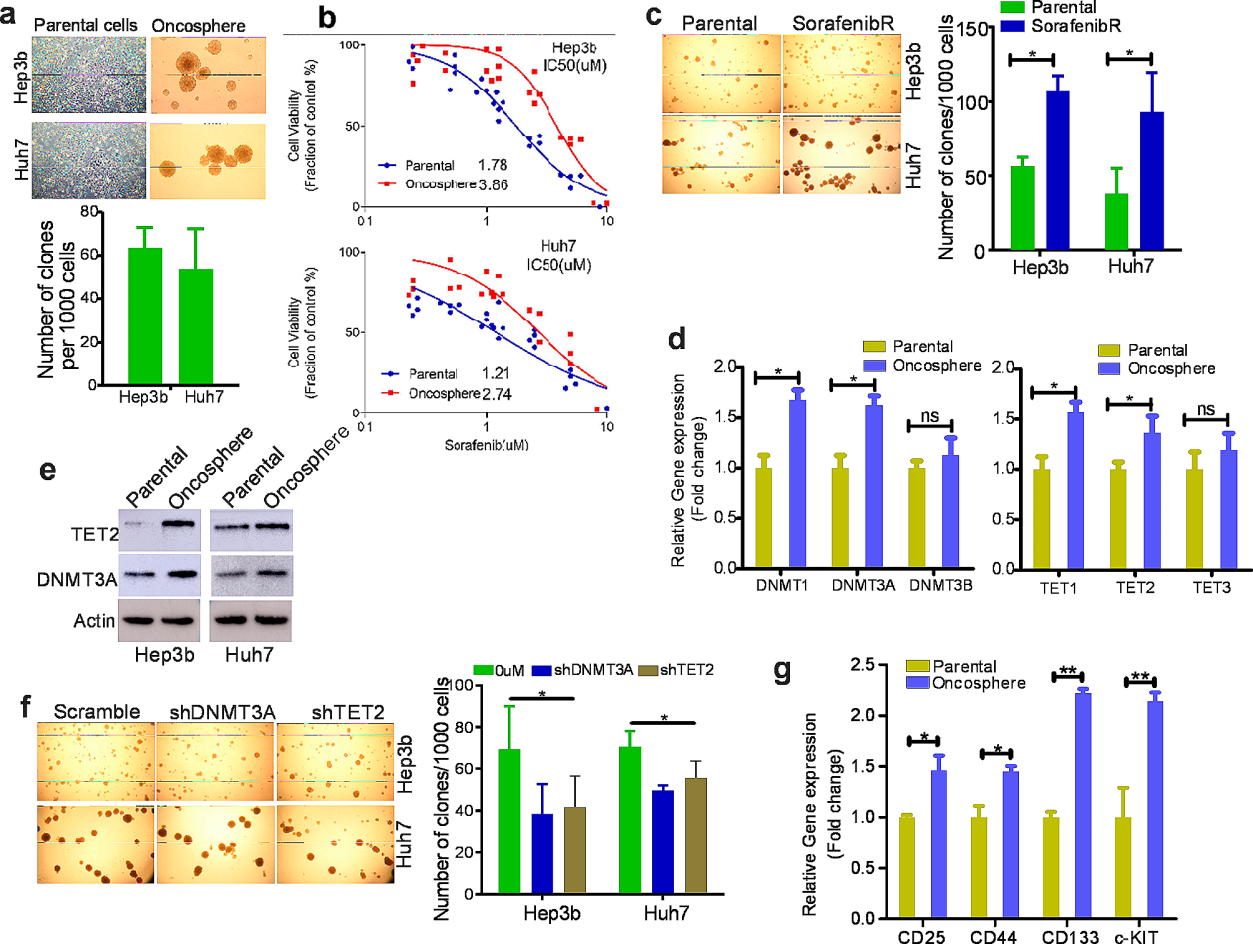
Role of stem cell-like cells in sorafenib resistance. (**a**), Oncosphere-forming assays in parental HCC cells. (**b**), CCK-8 assays in oncosphere and parental cells treated with indicated doses of sorafenib for 72 h. The data represent two independent experiments with 8 repeats in total. (**c**), Oncosphere-forming assays in parental and resistant HCC cells. (**d**), qPCR measuring the RNA expression of DNMTs and TETs in oncosphere and parental cells. (**e**), Western blot for the protein expression of DNMT3a and TET2 in oncosphere and parental cells. (**f**), Sorafenib^R^ Hep3b and Huh7 cells were infected with DNMT3a or TET2 shRNA or control viruses, and selected by 2 µg/ml puromycin for 5 days. Then these cells were subjected to oncosphere-forming assays. (**g**), qPCR for RNA expression of stem cell markers in oncosphere and parental cells. *p < 0.05; **p < 0.01; ns, not significant

Given highly heterogeneous HCC cells and the role of oncospheres in developing drug resistance, we performed oncosphere-forming assays in HCC Hep3b and Huh7 cells, and found that about 6% and 5% cells (ratio: Onco/total) can form oncospheres, respectively (Fig. 4a). To examine the drug sensitivity of oncospheres, we replated oncospheres and treated them with different doses of sorafenib. As shown in Fig. 4b, the replated oncosphere cells displayed IC_50_ values to sorafenib significantly larger than those exhibited by their parental counterparts, suggesting that oncospheres may at least partially mediate the development and maintenance of sorafenib resistance. In line with this, sorafenib^R^ Hep3b and Huh7 cells exhibited greater tendency to form oncospheres than parental cells (Fig. 4c). To identify essential regulators for oncosphere formation, we examined the levels of DNMTs and TETs. We found that both RNA and protein expression of DNMT3a and TET2 are highly elevated in oncospheres compared to parental cells (Fig. 4d and e). We used shRNA lentiviruses to knock down DNMT3a and TET2, and found that DNMT3a or TET2 inactivation inhibits oncosphere growth as supported by reduction of oncosphere number without obvious changes of oncosphere sizes (Fig. 4f). In addition, we found that oncosphere cells express elevated levels of stem cell makers like CD133, CD25, CD44 and c-KIT (Fig. 4g). Therefore, we propose that upregulation of DNMT3a and TET2 in CSCs is essential for the development of sorafenib resistance.

### Dysregulation of DNMTs and TETs is linked to survival and sorafenib responses in HCC patients

**Fig. 5:**
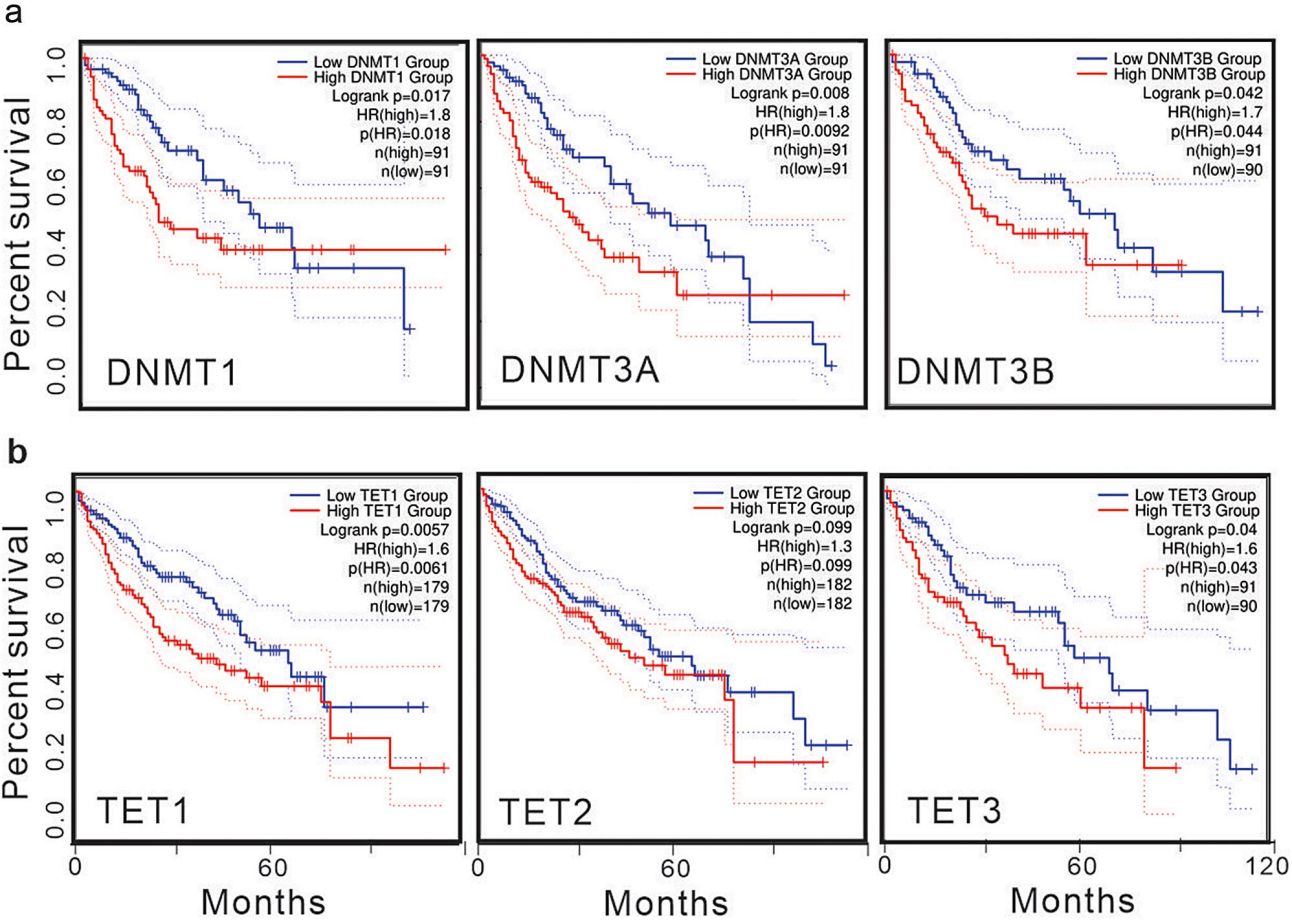
Upregulation of DNMTs and TETs in HCC patients predicts unfavorable outcomes. **a** and **b**, the association of DNMTs and TETs expression with survival in liver cancer patients, which were generated in online tool GEPIA2 (c; http://gepia.cancer-pku.cn/)

To validate the prognostic implications of DNMTs overexpression, we conducted a survival analysis of HCC patients using online tools (Gepia2) to examine the association between patient outcomes and DNMTs levels. We observed that HCC patients with higher expression levels of DNMT1, DNMT3a, and DNMT3b exhibited significantly shorter survival times compared to patients with lower levels (Fig. 5a). Similarly, employing the same methodology to validate the prognostic implications of TETs overexpression, we noted that HCC patients with elevated levels of TET1 and TET3 had markedly shorter survival times than those with lower levels (Fig. 5b). While the p-value was 0.099, indicating a marginal significance, there still existed a noticeable trend suggesting that HCC patients with higher TET2 expression tended to have shorter survival times than those with lower levels (Fig. 5b).

Subsequently, in order to elucidate the clinical ramifications of upregulated DNMTs and TETs in sorafenib-resistant HCC cells, we analyzed their mRNA levels in the public dataset GSE109211. Our findings revealed significant upregulation of DNMT1, DNMT3a, DNMT3b, TET1, and TET2 in sorafenib non-responders (*n* = 21) in comparison to responders (*n* = 40) (Fig. S3a and S3b) (No TET3 data). Evidently, sorafenib non-responding patients exhibited substantially shorter survival times than responders (Fig. S3c). Additionally, we examined the correlation between patient outcomes and the expression of DNMT3A and TET2 in sorafenib responders. We found that those with higher levels of DNMT3A and TET2 had a slightly lower survival probability compared to responders with lower expression,). Inhough the difference did not reach statistical significance (Fig. S3d). In summary, our data suggests that DNMT3A and TET2 play pivotal roles as regulators of sorafenib resistance in HCC.

### Pharmacological targeting of TET2 impairs sorafenib^R^ cell growt2

**Fig. 6:**
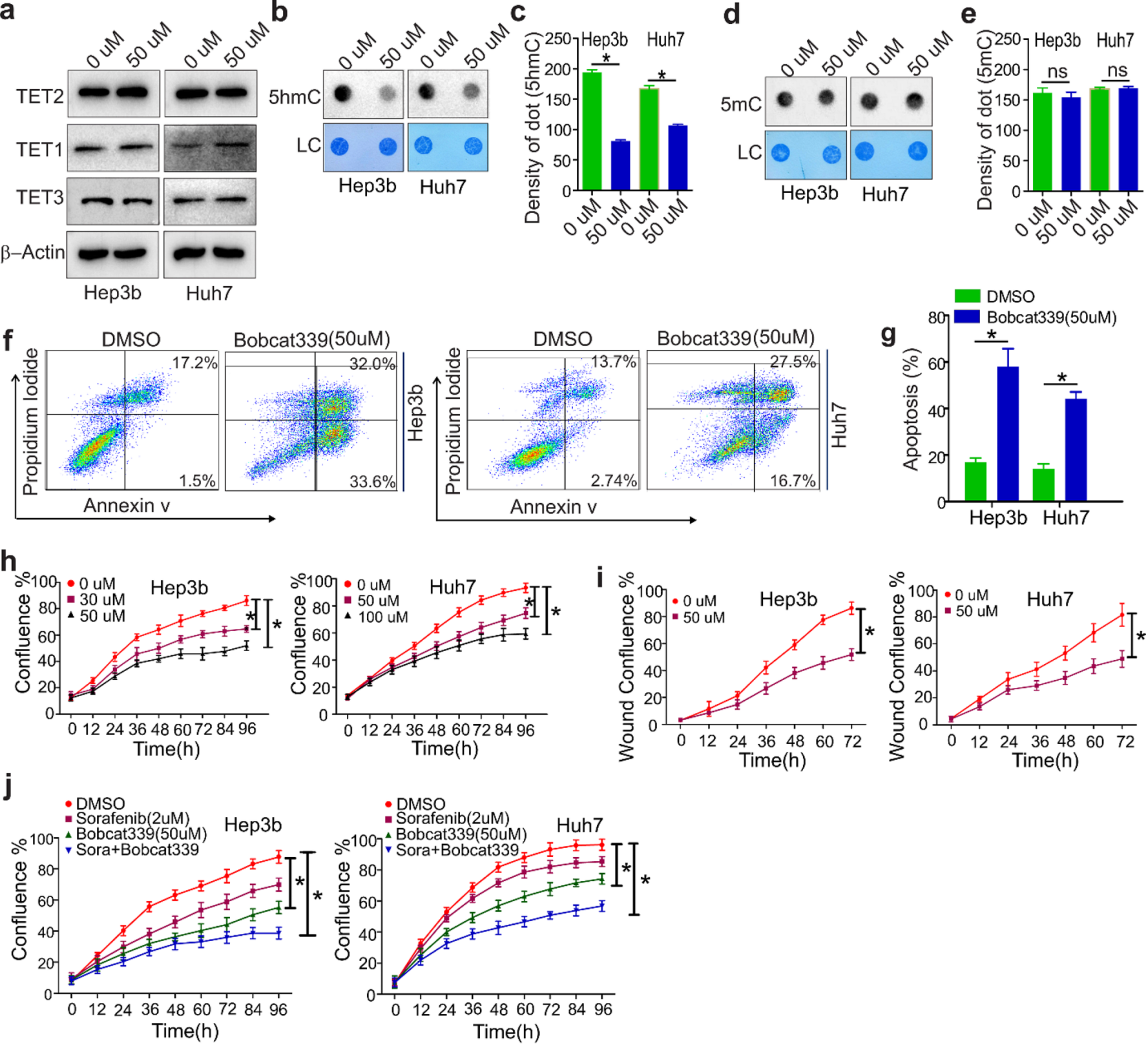
TET2 inhibitor Bobcat339 impairs resistant cell growth. a-e, Sorafenib^R^ Hep3b and Huh7 cells were treated with 50 µM TET2 inhibitor Bobcat339 for 72 h. (**a**), Western blot measuring protein expression of TET2, TET1 and TET3. Data represents three independent experiments. b and c, Dotblotting showing the changes of 5hmC amount (**b**). The graphs (**c**) are the quantification of dot intensity as mean values ± S.D. from three independent experiments. d and e, Dotblotting to assess changes in 5mC amount (**d**). The graphs (**e**) are the quantification of dot intensity as mean values ± S.D. from three independent experiments. f and g, Flow cytometry showing cell apoptosis (**f**). Graphs (**g**) are quantification of cell apoptosis (%) as mean values ± S.D. from three independent experiments. h and i, Proliferation (**h**) and migration (**i**) assays. Data represents two independent experiments with 12 repeats in total. (**j**), Sorafenib^R^ Hep3b and Huh7 cells were treated with either 50 µM Bobcat339 alone or plus 2 µM sorafenib for 72 h. Cell proliferation assays were used to determine drug response. Data represents two independent experiments with 12 repeats in total. *P < 0.05; LC, loading control

Inhibition of resistant cell growth from TET2 upregulation in sorafenibR cells and TET2 gene knockdown implies that TET2 could be a pharmacological target. To investigate this, we treated sorafenib^R^ Hep3b and Huh7 cells with bobcat339, a selective cytosine-based TET enzyme inhibitor [29]. Western blotting revealed that exposure to bobcat339 does not change protein expression of either TET2, TET1 and TET3 (Fig. 6a). Dotblotting analysis showed that bobcat339 treatment reduces DNA 5-hmC abundance (Fig. 6b and c) without obvious changes of 5mC amount (Fig. 6d and e), consistent with the concept that bobcat339 does not inhibit *de novo* methyltransferase DNMT3a [29]. 

To assess if exposure to bobcat339 inhibits sorafenib^R^ cell growth, we performed flow cytometry and observed that cell apoptosis is significantly increased in the presence of bobcat339 (Fig. 6f and g). Further, proliferation and wound-healing assays found that sorafenib^R^ cells treated with bobcat339 proliferate and migrate at a much lower rate than untreated cells (Fig. 6h and i; Fig. S4a and Fig. S4b). Additionally, the combination of sorafenib with bobcat339 resulted in more pronounced inhibition of sorafenib^R^ cell proliferation than either treatment alone (Fig. 6j; Fig. S4c). Collectively, these results support that TET2 inhibitors may be promising therapeutic reagents in overcoming sorafenib resistance, which merits thorough investigations in vivo.

### DNMT3a and TET2 regulate sorafenib sensitivity and coordinately modulate sorafenib^R^ cell proliferation

**Fig. 7:**
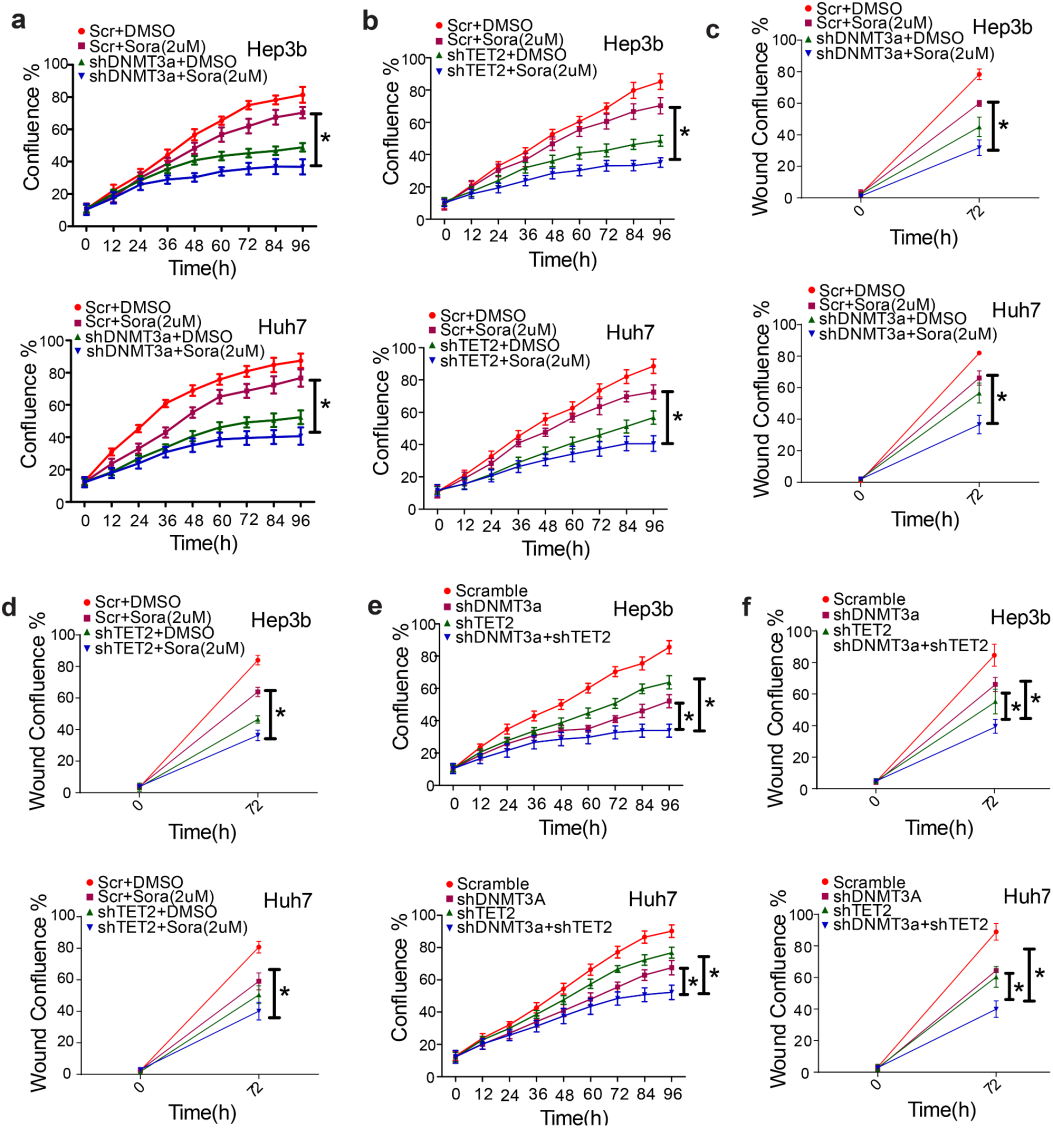
The impacts of DNMT3a and TET2 expression on sorafenib sensitivity. (**a** and **b**), Sorafenib^R^ Hep3b and Huh7 cells were infected with DNMT3a or TET2 shRNA or control viruses, and selected by 2 µg/ml puromycin for 2 days. Then these cells were exposed to 2 µM sorafenib followed by IncuCyte proliferation assays. (**c**) and (**d**), Sorafenib^R^ Hep3b and Huh7 cells were infected with DNMT3a or TET2 shRNA or control viruses, and selected by 2 µg/ml puromycin for 2 days. Then these cells were exposed to 2 µM sorafenib followed by wound-healing assays. (**e** and **f**), Sorafenib^R^ Hep3b and Huh7 cells were infected with either DNMT3a and TET2 shRNA viruses alone or both, selected by 2 µg/ml puromycin for 2 days and subjected to IncuCyte proliferation assays. Data represents two independent experiments with 12 repeats in total. *P < 0.05; Scr, Scramble; Sora, Sorafenib

Given that both DNMT3a and TET2 are upregulated in resistant cells, and as knockdown of DNMT3a or TET2 suppresses resistant cell growth, we speculated that DNMT3a or TET2 expression may be associated with sorafenib sensitivity. Therefore, DNMT3a or TET2 was knocked down in sorafenib^R^ Hep3b and Huh7 cells identified with DNMT3a or TET2 overexpression. These cells were treated with 2 µM of sorafenib for indicated time points. Cell proliferation assays revealed that depletion of DNMT3a or TET2 sensitizes sorafenib^R^ cells to sorafenib-inhibited cell proliferation (Fig. 7a and b). Wound-healing studies uncovered that DNMT3a or TET2 knockdown enhances sorafenib-impaired cell migration (Fig. 7c and d). These findings indicate that DNMT3a or TET2 expression is required to maintain sorafenib-resistant phenotypes of HCC cells.

Finally, to establish the role of DNMT3a and TET2 interaction on sorafenib^R^ cell growth, we knocked down either DNMT3a, TET2, alone or both. The cell proliferation assays revealed that concurrent knockdown of DNMT3a and TET2 leads to more robust inhibition of cell proliferation and migration than that of a single gene change (Fig. 7e and f. This suggests that DNMT3a and TET2 have functional cooperation in sustaining proliferation and migration of sorafenib^R^ HCC cells.

### DNMT3a physically interacts with TET2, but does not form a regulatory loop, to enhance sorafenib resistant phenotypes

Because DNMT3a and TET2 are upregulated simultaneously in sorafenib^R^ HCC cells, we first determined if DNMT3a regulates TET2 transcription or vice versa. By using online tools (GEPIA 2) and analyzing public data GDS4887, GSE45267 and GSE54236, we did not find an obvious correlation between DNMT3a and TET2 (Fig. S5a and S5b). These findings, together with observations that DNMT3a knockdown does not change TET2 protein expression or vice versa (Ref. Figure 3b and h), reflect the idea that DNMT3a and TET2 do not transcriptionally regulate each other. We then examined if DNMT3a and TET2 have protein interactions in HCC cells. Co-IP assays revealed that DNMT3a forms a complex with TET2 and HDAC2 in both sorafenib^R^ and parental HCC cells with a slight increase in their interaction potential in sorafenib^R^ cells (Fig. S5c and S5d). The protein interaction among DNMT3a, TET2 and HDAC2 was further confirmed by the results from string-interaction assays (Fig. S5e; https://string-db.org), which was consistent with previous studies showing that HDAC2 and TET2 are co-transcription factors of DNMT3a [30–32]. Given the dual function of TET2 in cancers, [32] these findings demonstrate that a transcriptional complex of DNMT3a, TET2 and HDAC2 exists in sorafenib^R^ HCC cells.

### Tumor suppressor genes are further silenced in sorafenib^R^ HCC cells by DNMT3a- mediated promoter DNA hypermethylation

**Fig. 8:**
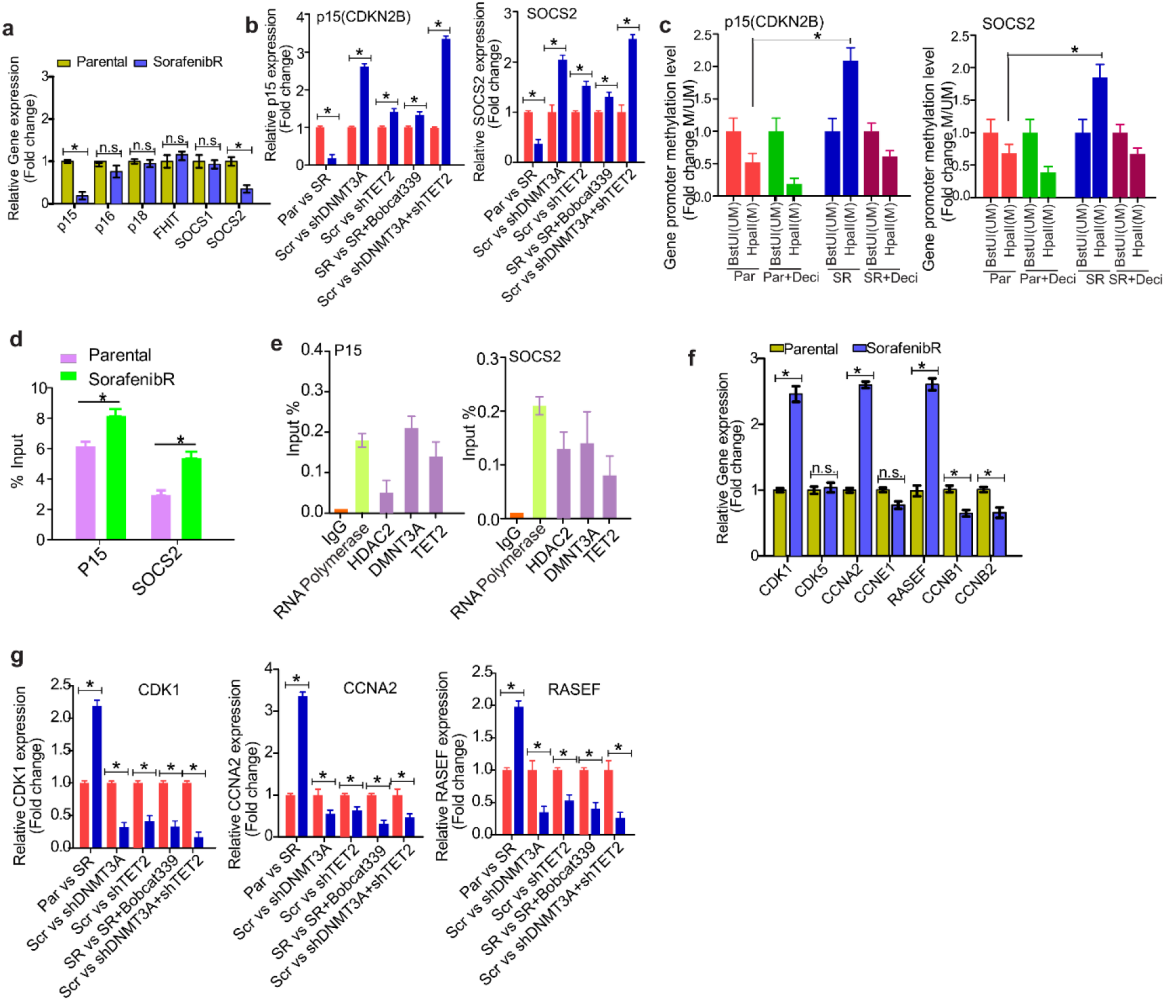
Gene regulation by DNMT3a and TET2 occurs in promoter DNA methylation -dependent and independent manners in sorafenib cells. (**a**), qPCR measuring the indicated TSG expression in Sorafenib^R^ Hep3b and Huh7 cells. (**b**), qPCR measuring the expression of p15 and SOCS2 in Sorafenib^R^ Hep3b cells either infected with DNMT3a or/and TET2 virus vectors or treated with bobcat339 for 48 h. (**c**), DNA (1 µg) from parental or Sorafenib^R^ Hep3b cells treated or untreated with 2 μm decitabine for 48 h were digested by HpaII or BstuI, and PCR was performed using primers specific for p15 or SOCS2 gene promoter. HpaII indicates no digestion, then hypermethylated; BstUI, no digestion, then hypomethylated. (**d**), MeDIP assays were performed in Sorafenib^R^ and parental Hep3b cells, and the 5mC-enriched DNA was subjected to qPCR using primers of p15 or SOCS2 gene promoter. (**e**), ChIP assays using antibodies for HDAC2, DNMT3a and TET2 were performed in Sorafenib^R^ Hep3b cells, and the ChIP-enriched DNA was subjected to qPCR using primers specific for p15 or SOCS2 promoter. (**f**), qPCR to measure the expression of CDK1, CCNA2 and RASEF in Sorafenib^R^ Hep3b and Huh7 cells. (**g**), qPCR to measure the expression of CDK1, CCNA2 and RASEF in Sorafenib^R^ Hep3b cells either infected with DNMT3a or/and TET2 virus vector or treated with bobcat339 for 48 h. Data represents three independent experiments. *P < 0.05; ns, not significant. SR, Sorafenib^R^; Scr, scramble; Par, parental; Deci, decitabine

To define the mechanisms in which DNA methylation aberrations contribute to faster growth of sorafenib^R^ cells, we examined the changes of epigenetically silenced tumor suppressor genes (TSGs) such as, p15, p16, p18, FHIT, SOCS1 and SOCS2. These TSGs are frequently silenced by promoter DNA methylation and their downregulation predicts worse prognosis in HCC patients. The results from qPCR showed that expression of p15 and SOCS2 is largely decreased in sorafenib^R^ cells compared with parental Hep3b and Huh7 cells, but no obvious changes were detected in the expression of p16, p18, FHIT, and SOCS1 (Fig. 8a). These results highlight that further TSG silencing is required for the survival and rapid proliferation of sorafenib^R^ cells.

As epigenetic regulators TET2 and DNMT3a are upregulated in sorafenib^R^ cells, we reasoned that overexpression of TET2 and DNMT3a may account for TSG silencing. To test this, we knocked down DNMT3a or disrupted TET2 activity via bobcat339 in sorafenib^R^ Hep3b and Huh7 cells, and assessed expression of these TSGs. As expected, DNMT3a ablation significantly increased the levels of p15 and SOCS2, but genetic or pharmacological inactivation of TET2 did not alter their expression. Notably, co-knockdown of TET2 and DNMT3a led to more pronounced upregulation of p15 and SOCS2 (Fig. 8b), suggesting that TET2 and DNMT3a coordinately silence TSGs in sorafenib^R^ cells.

To investigate the methylation status of p15 and SOCS2 promoters, genomic DNA from sorafenib^R^ and parental cells was digested with the restriction enzyme HpaII, which targets nonmethylated sites, or BstUI which cuts methylated sites. Decitabine-treated cells were used as positive controls. Following digestion, DNA was analyzed by qPCR using primers specific to p15 or SOCS2 gene promoter containing CpG islands. Efficient digestion by HpaII or BstuI led to stronger amplification in hypomethylated or hypermethylated p15 and SOCS2 promoters, respectively, in sorafenib^R^ cells compared to parental controls. Consistent with the silencing data (see Fig. 8a) and opposite to decitabine-treated controls, these data provide evidence that the p15 and SOCS2 promoters are more hypermethylated in sorafenib^R^ cells than that in parental controls (Fig. 8c). To precisely measure the alterations of promoter methylation, we performed MeDIP assays, in which methylated DNA was enriched by 5mC antibody and quantified by qPCR. Consistent with the findings from enzyme digestion (see Fig. 8c), the levels of 5mC in the promoters of p15 and SOCS2 were significantly increased in sorafenib^R^ cells compared to parental controls (Fig. 8d). To address how TSG promoters become hypermethylated, we performed ChIP assays, and found that both DNMT3a and TET2 bind the promoters of p15 and SOCS2 (Fig. 8e). These findings support the idea that DNMT3a and TET2 collaborate to form a protein complex, thereby amplifying promoter DNA hypermethylation. Additionally, HDAC2 plays a crucial role in further silencing tumor suppressor genes (TSGs).

### DNMT3a and TET2 activate cell proliferation genes through promoter binding in a DNA methylation-independent manner

To further understand how sorafenib^R^ cells more aggressively proliferate and migrate, we first examined the expression of certain oncogenes, specifically, CDK1, CCNA2 and RASEF. We found that these oncogenes are highly elevated in sorafenib^R^ cells compared to parental cells (Fig. 8f). These results suggest that, in addition to TSG silencing, oncogenic upregulation may contribute to the survival and faster proliferation of sorafenib^R^ cells. Second, inactivation of either TET2 or DNMT3a led to a decrease of oncogene expression, but we did not see a synergistic effect on oncogenic downregulation upon knockdown of both TET2 and DNMT3a (Fig. 8g). Third, the assays of enzymatic digestion from bulk DNA did not show obvious changes of DNA methylation in the promoters of oncogenes CDK1, CCNA2 and RASEF (Fig. S6a). Instead, MeDIP assays revealed a slight decrease of 5mC in the promoters of CDK1 and CCNA2 but not in RASEF (Fig. S6b). Fourth, ChIP analysis disclosed that DNMT3a, TET2 and HDAC2 are enriched in the promoters of oncogenes CDK1, CCNA2, and RASEF (Fig. S6c), although such binding did not increase the levels of DNA methylation in sorafenib^R^ cells. These findings strongly suggest that both DNMT3a and TET2 have DNA methylation-independent functions [33, 34]. 

### Knockdown or overexpression of P15 and SOCS2 influences sorafenib resistance in HCC cells

To investigate the impact on sorafenib resistance, we manipulated the expression of tumor suppressor genes (P15 or SOCS2) in liver cancer cell lines. Given the reduced expression of P15 and SOCS2 in drug-resistant liver cancer cell lines, we opted to knock down these genes in parental cells and overexpress them in drug-resistant cell lines.

Initially, parental Hep3b and Huh7 cells were transduced with P15 siRNA, SOCS2 siRNA, or scrambled vectors for 24 h. As depicted in Fig. S7a and S7c, the mRNA levels of P15 or SOCS2 significantly decreased in siRNA-treated cells compared to the control group. Subsequently, P15- or SOCS2-depleted cells underwent a proliferation assay with 2 μm sorafenib. The results revealed that cells with P15 or SOCS2 knockdown exhibited a markedly accelerated rate of proliferation in a time-dependent manner compared to their control counterparts (Fig. S7b and S7d). For sorafenib-resistant (SorafenibR) Hep3b and Huh7 cells, P15 OE or SOCS2 OE or PGIPZ vectors were introduced for 24 h. As illustrated in Fig. S7e and S7g, the mRNA levels of P15 or SOCS2 significantly increased in the overexpression (OE) group compared to the control group. Subsequently, P15- or SOCS2-overexpressing cells underwent a proliferation assay with 2 μm sorafenib. The findings demonstrated that cells with P15 or SOCS2 overexpression proliferated at a considerably slower rate in a time-dependent manner than their control counterparts (Fig. S7f and S7h). These results reinforce the concept that reduced expression of P15 and SOCS2 in sorafenib-resistant cells enhances cellular drug resistance.

## Discussion

Sorafenib remains one of the limited treatment options for HCC and has become the standard therapy for advanced HCC patients. However, clinical responses to sorafenib are often short-lived, and many patients acquire drug resistance and experience disease recurrence. These outcomes suggest that successful treatment of advanced HCC patients would require the discovery of new molecular rules of acquired sorafenib resistance and implementation of effective treatment regimens. In this study, we present evidence showing that (1) in resistant cells, DNMT3a and TET2 are upregulated, global and gene-specific DNA methylation is increased, and TSGs are further silenced, but cell proliferation genes are activated. CSCs with overexpression of DNMT3a and TET2 have higher potential to form oncospheres and have reduced sorafenib sensitivity; (2) clinically, upregulation of DNMT3a and TET2 predicts less favorable drug responses and worse prognosis in sorafenib-treated HCC patients; (3) mechanistically, we proved that the DNMT3a-TET2 complex binds target promoters and coordinately regulate TSGs and cell proliferation genes via DNA methylation-dependent or -independent manners (Fig. S8). Our work uncovers a critical DNMT3a-TET2 coordination that drives HCC resistance to sorafenib, which launches a new era of discovery in the molecular biology of cancer drug resistance. Our findings suggest that coordinated silencing of DNMT3a and TET2 is an effective approach to treat refractory or relapsed HCC patients.

Given that sorafenib targets different signaling pathways, there could be multiple molecular mechanisms underlying acquired resistance. We focused on aberrant epigenetics/DNA methylation because (1) genetic mutations fail to explain why TKI (i.e., sorafenib) resistant phenotypes are partially reversible; [9, 20, 35] (2) under TKI selection, epigenetic changes accumulate as the cell population evolves and diversifies [36] at higher rates than genetic alterations. Furthermore, changes in resistant phenotypes and epigenetic modifications are both dynamic and reversible; [37–42] (3) our investigation supported data from multiple other studies showing that aberrant epigenetics (i.e., DNA methylation) significantly contributes to TKI resistance; [9, 20, 38] (4) while abnormal DNA methylation indeed has been appreciated in sorafenib resistance, the regulatory and functional roles of DNA methylation regulators are still barely defined. As HCC is highly heterogeneous, we speculated that, upon exposure to sorafenib, the dynamic and reversible traits of DNA methylation allow TSGs to be rapidly and further epigenetically silenced, or oncogenes to be activated, which helps maintain the survival and proliferation of a subpopulation of cells through sorafenib killing. Indeed, our studies identified a previously unknown molecular rule, a DNMT3a-TET2 crosstalk, in determining HCC cell fate when facing sorafenib-imposed selective pressure. First, CSCs have DNMT3a and TET2 upregulation, higher potential to form oncospheres, and increased tolerance to sorafenib. These CSCs could survive and proliferate, leading to a resistant population. Second, the levels of DNMT3a, TET2, and DNA methylation are highly elevated in resistant vs. parental cells. As a consequence, TSGs (i.e., P15, SOCS2) are further silenced and oncogenes (i.e., CDK1, CCNA2, and RASEF) are activated. Third, HCC patients with higher DNMT3a and TET2 levels have a less favorable response to sorafenib, and a shorter survival time, consistent with previous investigations [43, 44]. Fourth, disruption of DNMT3a-TET2 crosstalk impairs resistant growth and sensitizes sorafenib^R^ cells to sorafinib treatment, where TSGs are re-expressed through promoter DNA hypomethylation and oncogenes are downregulated. Finally, concurrent dysfunction of DNMT3a and TET2 leads to more robust inhibition of sorafenib^R^ cell growth than single gene changes. Thus, DNMT3a-TET2 crosstalk serves as a new prognostic biomarker and a novel therapeutic target in overcoming HCC sorafenib resistance.

When DNA is methylated by DNMTs, 5mC can be the substrate of TET-mediated oxidation process that forms 5hmC, leading to DNA demethylation [8]. Thus, aberrant TET activities by gene dysregulation or mutations significantly regulates cancer pathogenesis and drug resistance. Of these three TETs, TET2 frequently obtains loss-of-function mutations, which greatly influence leukemic disease, [45] but have limited impacts on solid tumors including HCC [46]. Although the tumor suppressor role of TETs followed by increased 5hmC has been appreciated, our findings revealed that TETs are upregulated or have a trend toward upregulation in HCC compared to normal tissues. HCC patients with higher TETs have sorter life expectancy than those with lower TETs. These findings are consistent with recent findings that TET2 is highly expressed in HCC tissues, and TET2 overexpression is positively correlated with a shorter survival time, although TET1 upregulation plays an oncogenic role in other cancers, like leukemia [14] and breast cancer [15]. Regardless, one uniqueness of our studies is the demonstration that TET2 is an important contributor to acquired sorafenib resistance, because TET2 is significantly upregulated in sorafenib^R^ HCC cells, and a poorer survival is noticed in sorafenib-treated HCC patients overexpressing TET2. TET2 knockdown or treatment with bobcat339 impairs sorafenib^R^ cell growth, and partially restores sorafenib sensitivity. Given the upregulation of TET1 and TET3, the studies whether TET1 and TET3 are essential for sorafenib resistance and how TETs are upregulated in sorafenib^R^ cells are warranted.

While it is well known that TET2 converts 5mC into 5hmC, few studies have revealed a negative correlation between 5mC and 5hmC abundance in cancer cells. Our study showed that both TET and DNMTs are upregulated, and concomitantly high levels of global 5mC and 5hmC co-exist in sorafenib^R^ cells compared with parental counterparts. Nonetheless, it remains unclear whether the net outcomes of DNA methylation are more related to 5mC or 5hmC in sorafenib^R^ cells. While our promoter methylation assays revealed that certain TSGs have DNA hypermethylation in their promoter, further genome-wide DNA methylation analysis is required to define the re-distribution of 5mC or 5hmC and which genes or regions have a net outcome of 5mC or 5hmC enrichment. These thorough investigations will identify new epigenetic biomarkers for diagnosis and therapeutics in overcoming sorafenib resistance.

Finally, both DNMT3a and TET2 have DNA methylation-independent functions, serving as a scaffold protein that recruits other transcription factors to silence target gene expression. For example, DNMT3a interacts with AML1-ETO to regulate the expression of miR-193, [47] and binds HDAC to silence gene expression [48]. Further, TET2 forms a complex with DNMT1 or p300 to modulate DNA methylation in certain gene promoters [34]. However, such methylation-independent functions of DNMT3a and TET2 in sorafenib resistance are not defined. We demonstrate that while DNMT3a and TET2 are upregulated, they do not form a regulatory loop, because there is no positive correlation of their expression. Knockdown of TET2 did not change the DNMT3a expression or vice versa, rather, TET2 forms a complex with DNMT3a and HDAC2 in sorafenib^R^ cells and this complex binds TSG promoters to further silence TSGs in a DNA methylation-dependent manner (Fig. S8). In contrast, DNA methylation is not increased in the promoters of oncogenes CDK1, CCNA2, and RASEF, although both DNMT3a and TET2 bind these genes’ promoters. These findings are in consistent with the concept that upregulation of the aforementioned oncogenes is attributed to the DNMT3a-TET2-HDAC2 complex in a DNA methylation-independent manner in resistant cells (Fig. S8). Not only do these findings identify a novel complex, DNMT3a/TET2/HDAC2, in sustaining sorafenib^R^ cell growth, but also these results provide a mechanistic explanation for a new functional cooperation of DNMT3a and TET2 in developing sorafenib resistance.

## Conclusions

Our study uncovers a coordinated DNMT3a-TET2 in response to sorafenib as a hitherto unknown molecular base to initiate and sustain HCC resistance. Our data demonstrates a new mechanistic rule of aberrant epigenetic regulators in determining HCC cell fate under sorafenib-imposed selective pressure, in which the DNMT3a-TET2-HDAC2 complex modulates TSGs and cell proliferation genes through DNA methylation-dependent and -independent manners. Our results suggest an attractive prognostic biomarker and novel therapeutic target (DNMT3a-TET2-HDAC2 complex) for refractory HCC patients. Thus, our findings warrant further exploration of targeting the DNMT3a-TET2 coordination as an alternative approach to counteract sorafenib resistance. Given that there is limited efficacy of available inhibitors for epigenetic abnormality in HCC therapy and there exists DNMT3a-TET2 coordination in CSCs, disruption of DNMT3a-TET2 interplay could be used in a neo-adjuvant manner to prevent emergence of resistant oncospheres or eradicate sorafenib-resistant cells.

### Electronic supplementary material

Below is the link to the electronic supplementary material.

Supplementary Material 1

## Data Availability

The analyzed datasets in the present study are available from the corresponding author on reasonable request.

## List of abbreviations

CSCs: Cancer stem-like cells
Co-IP: Co-immunoprecipitation
CCK-8: Cell counting Kit-8
ChIP: Chromatin immunoprecipitation
DMSO: Dimethyl sulfoxide
GEO: Gene expression omnibus
HCC: Hepatocellular carcinoma
5hmC: 5-Hydroxymethylcytosine
5mC: 5-methylcytosine
DNMT1: DNA methyltransferase 1
DNMT3a: DNA methyltransferase 3a
DNMT3b: DNA methyltransferase 3b
FHIT: Fragile Histidine triad diadenosine triphosphatase
HDAC2: Histone deacetylase 2
MeDIP: Methylated DNA immunoprecipitation
PDGFRA: Platelet derived growth factor receptor alpha
PMSF: Phenylmethane sulfonyl fluoride
RASSF1: Ras association domain family member 1
SOCS1: Suppressor of cytokine signaling 1
SOCS2: Suppressor of cytokine signaling 2
Sorafenib^R^: Sorafenib resistant cells
TET1: Ten-eleven translocation methylcytosine dioxygenase 1
TET2: Ten-eleven translocation methylcytosine dioxygenase 2
TET3: Ten-eleven translocation methylcytosine dioxygenase 3
TSGs: Tumor suppressor genes
VEGF: Vascular endothelial growth factor
