# Supplementary material for: Coordinated activation of DNMT3a and TET2 in cancer stem cell-like cells initiates and sustains drug resistance in hepatocellular carcinoma
Source: Cancer Cell Int. 2024 Mar 25;24:110. doi: 10.1186/s12935-024-03288-3 (PMC10962188; doi:10.1186/s12935-024-03288-3)
Supplement: Supplementary file 1 — Supplementary Material 1 [file 12935_2024_3288_MOESM1_ESM.docx]

**Supplementary Information**

**Coordinated activation of DNMT3a and TET2 in cancer stem cell-like cells initiates and sustains drug resistance in hepatocellular carcinoma**

Tao Cheng, Changli Zhou, Sicheng Bian, Kelsey Sobeck, Yahui Liu

**Inventory of Supplemental Information**

**Supplemental Figures and Legends**

- **Figure S1, Related to Figure 1**
- **Figure S2, Related to Figure 3**
- **Figure S3, Related to Figure 5**
- **Figure S4, Related to Figure 6**
- **Figure S5**
- **Figure S6, Related to Figure 8**
- **Figure S7**
- **Figure S8, Schematic model**

**Supplemental Tables**

**Supplementary Table 1: Sequence of primers used in the experiments**

**Supplementary Table 2: Antibodies used in the experiments**


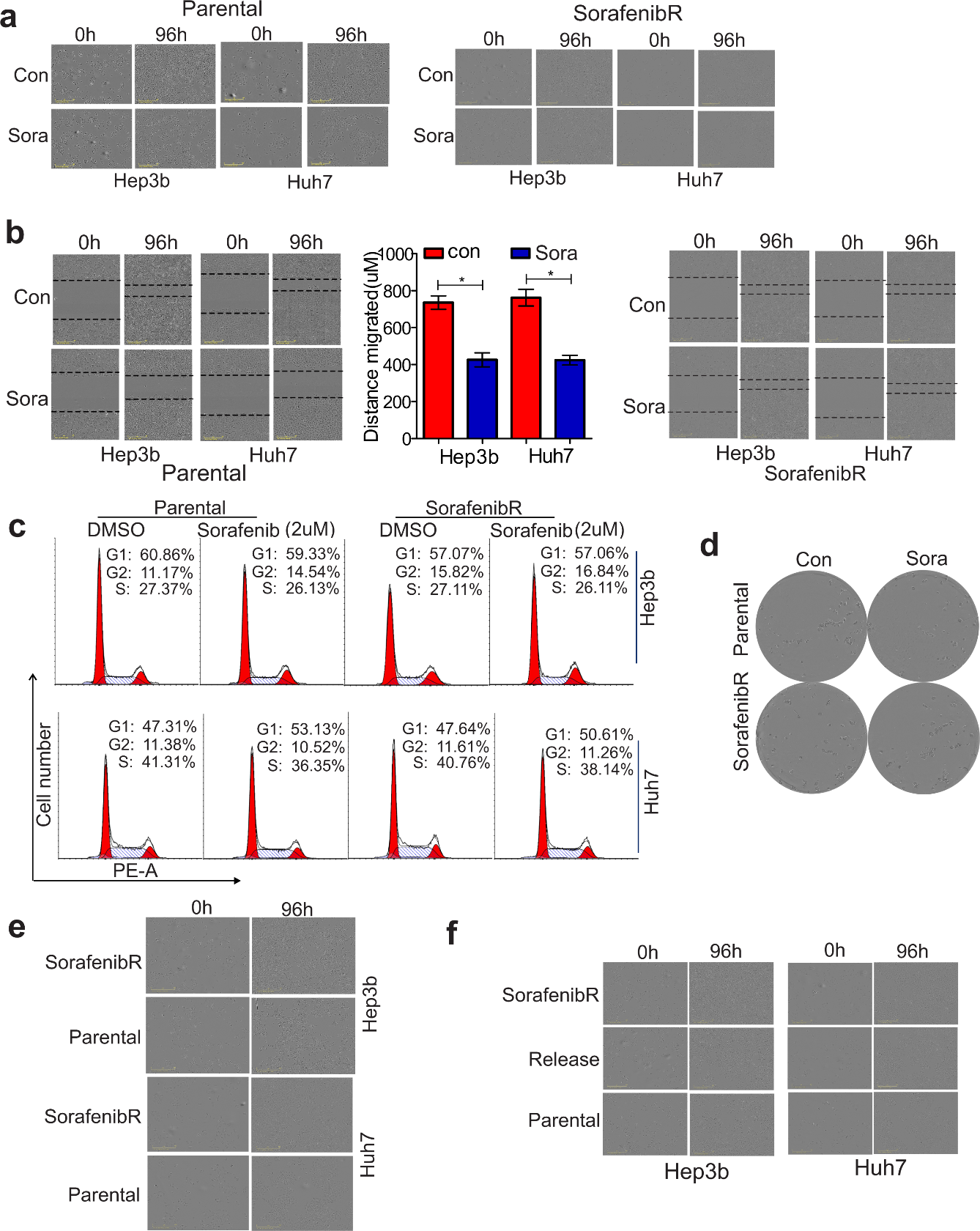


**Supplementary Figure 1. Characterization of sorafenib resistant cells. a** and **b**, Resistant or parental Hep3b or Huh7 cells were transiently treated with 2 µM sorafenib and subjected to proliferation (**a**) and wound-healing (**b**) assays. Images show the status of cell growth at 96 hours post-drug treatment, and represent two independent experiments with 12 repeats in total. Graphs (**b**, middle panel) show the migrate distance at 96 hours post-treatment shown as mean values ± S.D. from three independent experiments. **c**, Flow cytometry for cell cycle in sorafenib^R^ or parental Hep3b or Huh7 cells treated with 2 µM sorafenib for 72 hours. Data represent three independent experiments. **d**, Oncosphere assays in parental or resistant cells with or without drug treatment. **e**, IncuCyte proliferation assay in parental and resistant Hep3b or Huh7 cells growing in drug free medium. **f**, IncuCyte proliferation assays in parental, resistant or released (resistant cells growing in drug free medium for 14 days) Hep3b or Huh7 cells treated with 2 µM sorafenib for 96 hours. **P* < 0.05; Con, Control; Sora, Sorafenib.


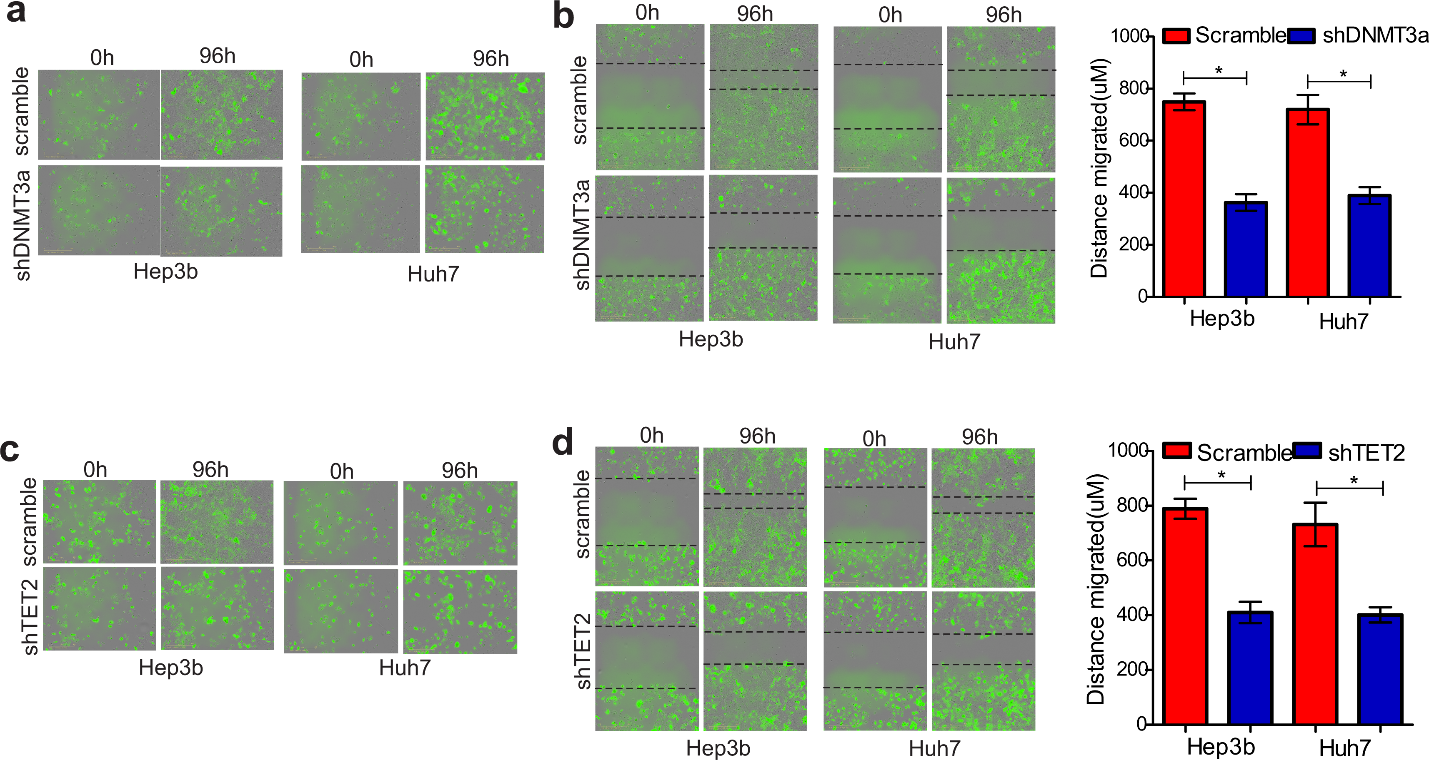


**Supplementary Figure 2. Expression of DNMT3a and TET2 is associated with Sorafenib^R^ cell growth. a** and **b**, Sorafenib^R^ Hep3b or Huh7 cells were infected by DNMT3a shRNA or control virus, selected by 2 ug/ml puromycin for 5 days in drug-free medium and subjected to proliferation (**a**) and wound-healing (**b**) assays. **c** and **d**, Sorafenib^R^ Hep3b or Huh7 cells were infected by TET2 shRNA or control virus, selected by 2 ug/ml puromycin for 5 days in drug-free medium and subjected to proliferation (**c**) and wound-healing (**d**) assays. Images show the status of cell growth at 96 hours post-drug treatment, and represent three independent experiments. Graphs show the migrate distance at 96 hours post-treatment shown as mean values ± S.D. from three independent experiments. **P* < 0.05.

**Supplementary Figure 3. Association of DNMT and TET expression with HCC patient drug response and survival. a,** Normalized expression of DNMT1, DNMT3a and DNMT3b in non-responder and responder HCC patients. **b**, Normalized expression of TET1 and TET2 in non-responder and responder HCC patients. **c**, the overall survival in non-responding and responding HCC patients analyzed by Log-rank test. **d**, the association of DNMT3a or TET2 expression with overall survival in liver cancer patients analyzed by Log-rank test. **p* < 0.05; ns, not significant.


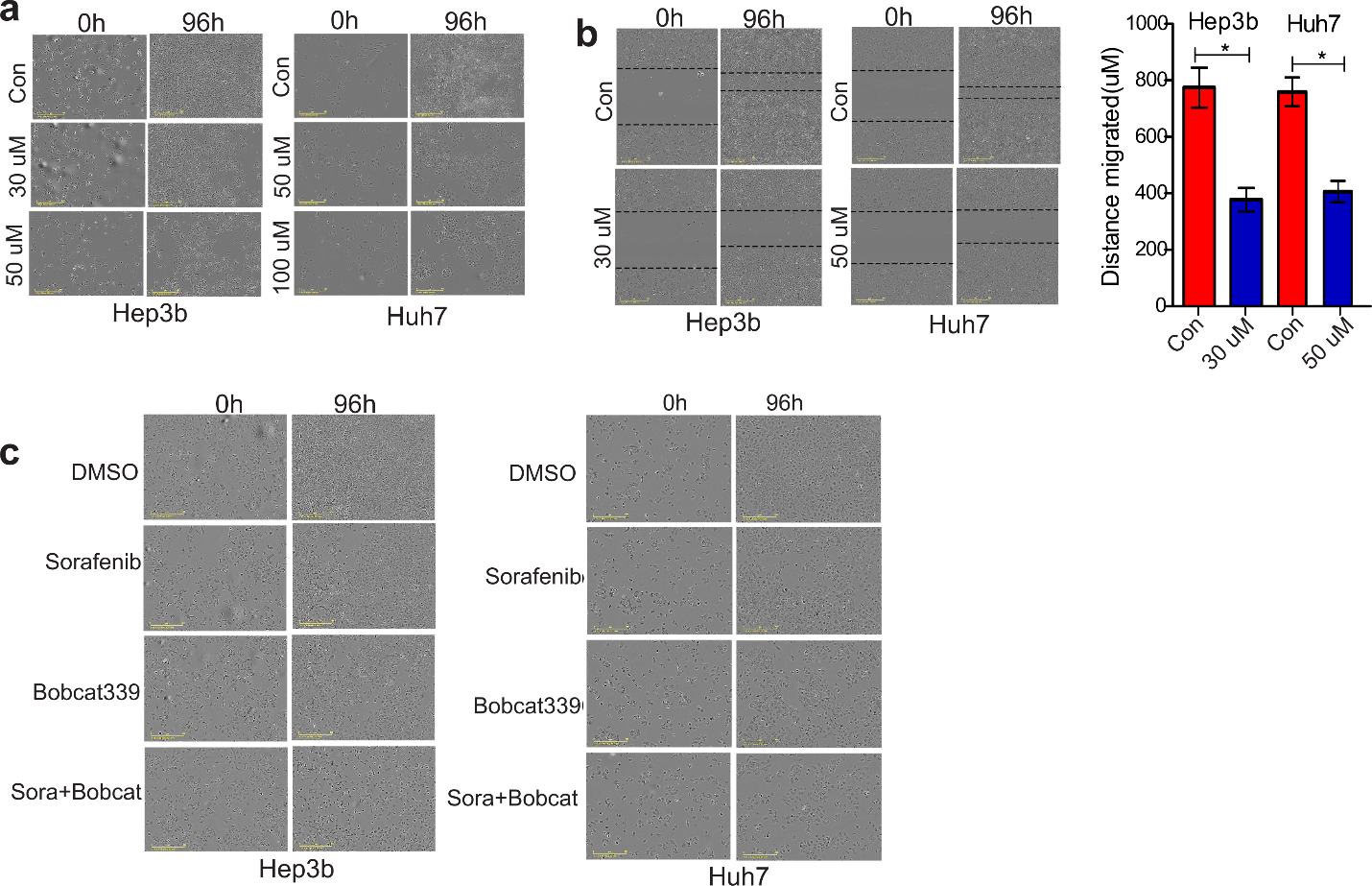


**Supplementary Figure 4. Treatment with bobcat339 inhibits resistant cell growth. a** and **b**, Sorafenib^R^ Hep3b or Huh7 cells were treated with indicated doses of bobcat339 and subjected to proliferation (**a**) and wound-healing (**b**) assays. **c**, Sorafenib^R^ Hep3b or Huh7 cells were treated with indicated doses of either bobcat339, sorafenib alone or both, and subjected to proliferation assays. Images show the status of cell growth at 96 hours post-drug treatment, and represent three independent experiments. Graphs are the quantification of the migrated distance. **P* < 0.05; Con, Control; Sora, Sorafenib.


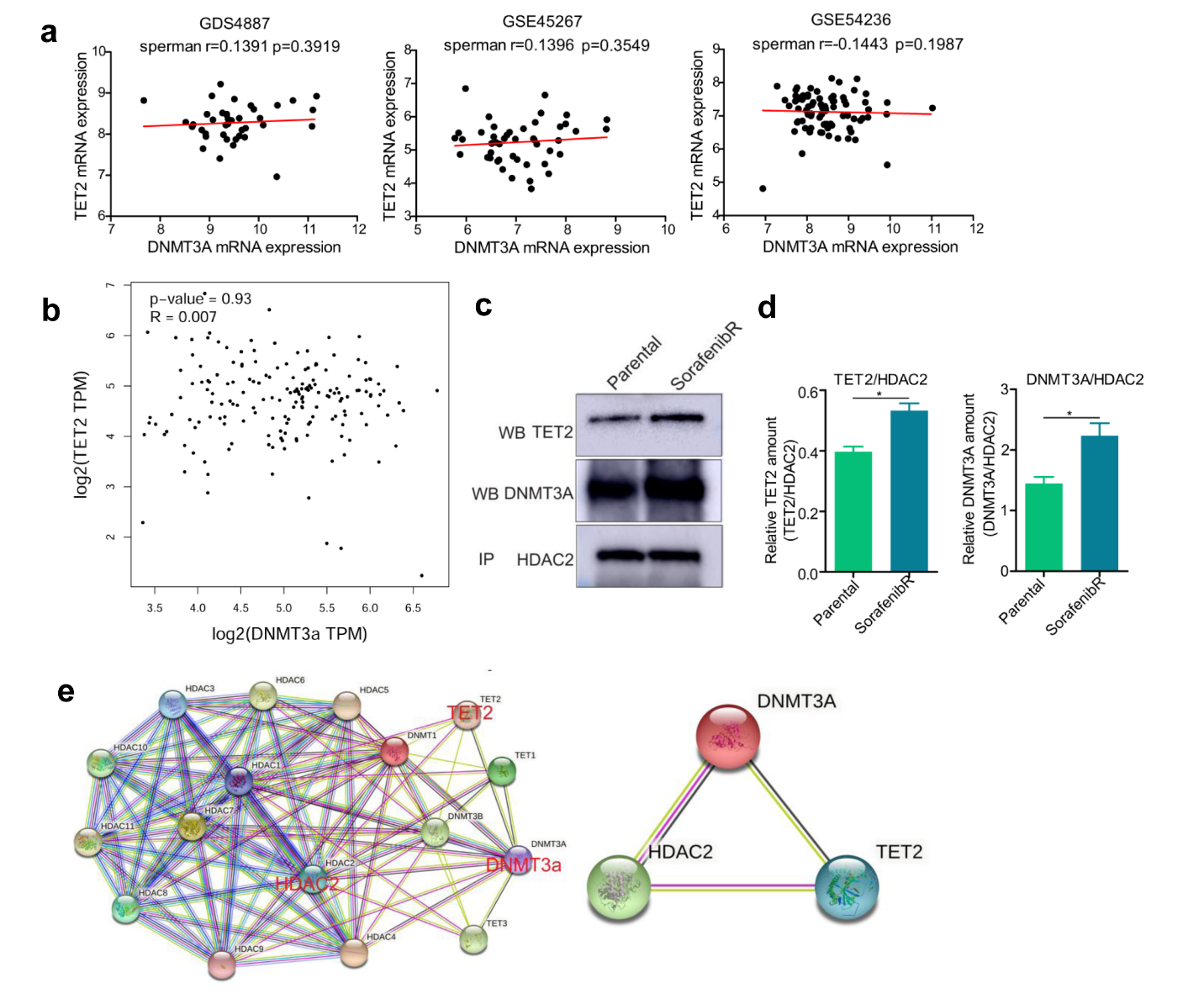


**Supplementary Figure 5. The regulatory interaction between DNMT3a and TET2 in HCC cells. a** and **b**, Correlation analysis between DNMT3a and TET2 mRNA levels in HCC patients from public datasets (**a**) or online tool (GEPIA 2; **b**). **c** and **d**, Co-immunoprecipitation assays for the interaction among DNMT3a, TET2 and HDAC2 in parental and sorafenib resistant Hep3b cells. Images (**c**) are representative from three independent experiments. Graphs (**d**) are the quantification of Western blot band intensity and a comparison of parental and resistant cells. **e**, Interaction-string assays for the indicated epigenetic regulators (left panel) and DNMT3a, TET2 and HDAC2 only (right panel). **P* < 0.05; IP, immunoprecipitation; WB, Western blot.


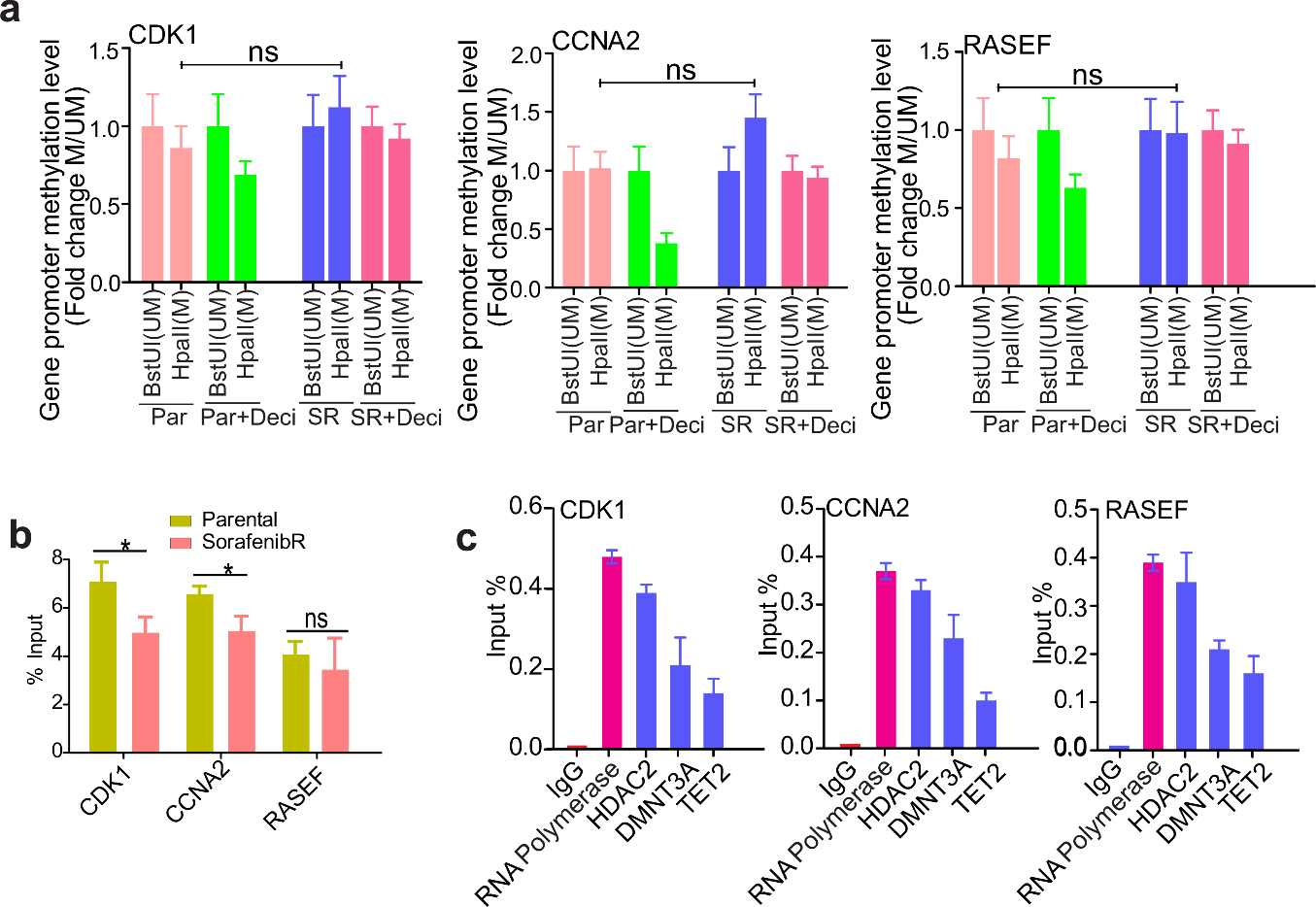


**Supplementary Figure 6. Promoter methylation of oncogenes is not obviously changed in Sorafenib^R^ compared to parental cells. a**, Genomic DNA from Sorafenib^R^ and parental cells were digested by HpaII or BstuI, and qPCR was performed using primers specific for the promoter of CDK1, CCNA2, and RASEF genes. Genomic DNA from parental cells treated with 2 uM decitabine for 48 hours was used as a positive control. HpaII indicates no digestion, then hypermethylated; BstuI, no digestion, then hypomethylated. **b**, MeDIP assays for promoter DNA methylation of CDK1, CCNA2 and RASEF genes in Sorafenib^R^ and parental Hep3b cells. **c**, ChIP assays using antibodies for HDAC2, DNMT3a and TET2 in Sorafenib^R^ Hep3b cells, and qPCR for the quantification of the ChIP-enriched DNA using primers specific for CDK1, CCNA2 and RASEF genes. Data represents three independent experiments. **P* < 0.05; ns, not significant; SR, Sorafenib; Par, Parental; Deci, Decitabine.

**Supplementary Figure 7.** **Knockdown or overexpression of P15 and SOCS2 influences sorafenib resistance in HCC cells.** a and c. qPCR results demonstrating the effective knockdown of P15 or SOCS2; b and d. Knockdown of P15 or SOCS2 resulting in an elevated level of drug resistance in liver cancer cells; e and g. qPCR results showcasing successful overexpression of P15 or SOCS2; f and g. Overexpression of P15 or SOCS2 leading to a reduction in drug resistance in liver cancer cells. (The concentration of sorafenib used in cell proliferation experiments is 2uM.


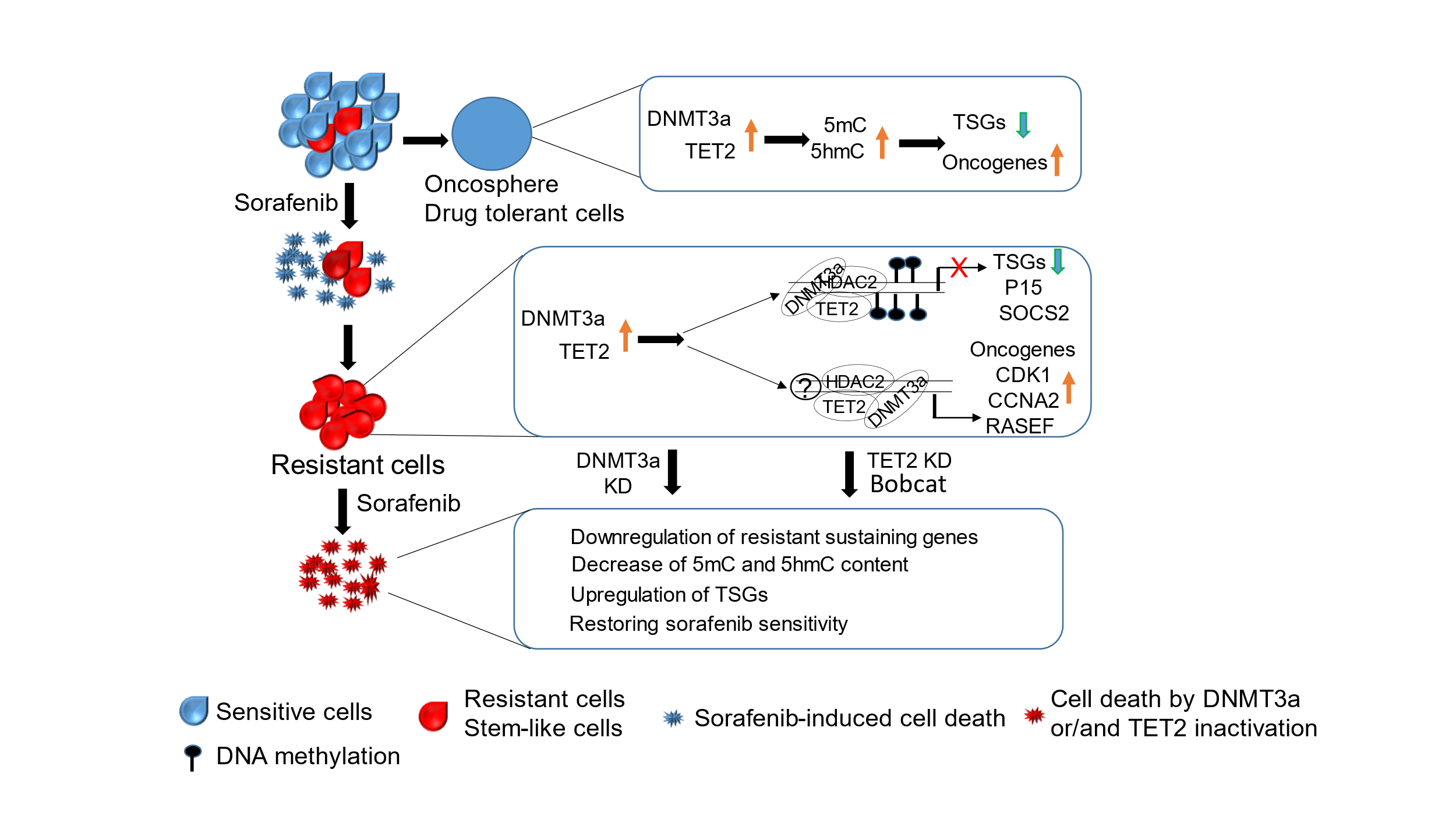


**Supplementary Figure 8. Schematic model illustrating the role of the DNMT3a and TET2 coordination in the acquired resistance to sorafenib.**

**Supplementary Table 1: Sequence of primers used in the experiments**

| Name (GenBank Accession No.) | | Primer Sequence (5' to 3') |
| --- | --- | --- |
| Primers for gene expression | |  |
| DNMT1(NM_001130823) | Forward | TATCCGAGGAGGGCTACCTG |
|  | Reverse | ATGAGCACCGTTCTCCAAGG |
| DNMT3A(NM_175629) | Forward | GCCATACGGTGGAGCCAT |
|  | Reverse | TGTTGAGCCCTCTGGTGAAC |
| DNMT3B(NM_006892) | Forward | GGAGATTCGCGAGCCCAG |
|  | Reverse | CTTCATGCTTTCCTGCCGC |
| TET1(NM_030625) | Forward | CAGAACCTAAACCACCCGTG |
|  | Reverse | TGCTTCGTAGCGCCATTGTAA |
| TET2(NM_001127208) | Forward | GGCTACAAAGCTCCAGAATGG |
|  | Reverse | AAGAGTGCCACTTGGTGTCTC |
| TET3(NM_001287491) | Forward | TCCAGCAACTCCTAGAACTGAG |
|  | Reverse | AGGCCGCTTGAATACTGACTG |
| P15(NM_004936) | Forward | GGGACTAGTGGAGAAGGTGC |
|  | Reverse | CATCATCATGACCTGGATCGC |
| SOCS2(NM_003877) | Forward | GTAGGGTAGAGGTGCCGAGA |
|  | Reverse | GGATGACAAAGTCCCTCGCA |
| CDK1(NM_001786.5) | Forward | GGCTCTTGGAAATTGAGCGG |
|  | Reverse | GGTATGGTAGATCCCGGCTT |
| CCNA2(NM_001237.5) | Forward | TGGTGGTCTGTGTTCTGTGAA |
|  | Reverse | AAACTTCTTGGATGCCAGTCT |
| RASEF (NM_152573.4 ) | Forward | GAACCGTCGTCGAAGGGAG |
|  | Reverse | TTCGGCCACTTGAGGGAAC |
| Primers for promoter DNA methylation |  |  |
|  |  |  |
| P15/CDKN2B(NG_023297) | Forward | TGGTGCTAGTAAGCGCGAAT |
|  | Reverse | TGAGAATCTTGCACGAGGCA |
| SOCS2(NC_000012.12) | Forward | GAGCCGGAGAGTCTGGTTTC |
|  | Reverse | TTCCTCTAAACATCCCGGCG |
| CDK1(NG_029877) | Forward | TTTCTTTCGCGCTCTAGCCA |
|  | Reverse | CAATCGGGTAGCCCGTAGAC |
| CCNA2(NG_052974) | Forward | ACACAGAGTCGGGGGATCTT |
|  | Reverse | CTCTTCACTTGAAGCACGCTG |
| RASEF(NC_000009.12) | Forward | ATGCGTTAACGAAAGCCAGT |
|  | Reverse | GGGGTAGGATTCCGAATGGG |
|  |  |  |

**Supplementary Table 2: Antibodies used in the experiments**

| Antibody | Application | Company | Catalog No. | Source | Dilution |
| --- | --- | --- | --- | --- | --- |
| DNMT1 | Western blot | Abcam | ab13537 | Rabbit | Western blot: 1:1000 |
| DNMT3a | Western blot  ChIP  Co-IP | Santa Cruz Biotechnology | sc20703 | Rabbit | Western blot: 1:1000  ChIP: 5 µg  Co-IP: 5 µg |
| DNMT3b | Western blot | Abcam | ab13604 | Rabbit | Western blot: 1:1000 |
| TET1 | Western blot | Santa Cruz Biotechnology | sc-163443 | Rabbit | Western blot: 1:500 |
| TET2 | Western blot  ChIP | Sigma-Aldrich | MABE462 | Rabbit | Western blot: 1:1000  ChIP: 5 µg |
| TET3 | Western blot | Abcam | ab139311 | Rabbit | Western blot: 1:1000 |
| β-actin | Western blot | Santa Cruz Biotechnology | sc-69879 | Mouse | Western blot: 1:1000 |
| HDAC3 | Western blot | Santa Cruz Biotechnology | sc-376957 | Mouse | Western blot: 1:1000 |
| HDAC2 | Western blot  ChIP  Co-IP | Abcam | ab7029 | Rabbit | Western blot: 1:1000  ChIP: 5 µg  Co-IP: 5 µg |
| 5mC | Dotblotting | Active Motif | 39649 | Rabbit | Dotblotting: 1:2500 |
| 5hmC | Dotblotting | Active Motif | 39069 | Rabbit | Dotblotting: 1:2500 |
| Anti-Rabbit | Western blot | Cell Signaling  Technology | 7074S | Goat | Western blot: 1:10000 |
| Anti-Mouse | Western blot | Cell Signaling  Technology | 7076S | Horse | Western blot: 1:10000 |
